# Comparative Anatomy of the Nasal Cavity in the Common Dolphin *Delphinus delphis* L., Striped Dolphin *Stenella coeruleoalba* M. and Pilot Whale *Globicephala melas* T.: A Developmental Study

**DOI:** 10.3390/ani11020441

**Published:** 2021-02-08

**Authors:** Alvaro García de los Ríos y Loshuertos, Marta Soler Laguía, Alberto Arencibia Espinosa, Alfredo López Fernández, Pablo Covelo Figueiredo, Francisco Martínez Gomariz, Cayetano Sánchez Collado, Nuria García Carrillo, Gregorio Ramírez Zarzosa

**Affiliations:** 1 Departamento de Anatomía y Anatomía Patológica Comparadas, Facultad de Veterinaria, Universidad de Murcia, 30100 Murcia, Spain; agrios@ceuta.es (A.G.d.l.R.yL.); f.gomariz@colvet.es (F.M.G.); scollado@um.es (C.S.C.); 2 Centro de Estudio y Conservación de Animales Marinos (CECAM), 51002 Ceuta, Spain; 3 Departamento de Medicina y Cirugía Animal, Facultad de Veterinaria, Universidad de Murcia, 30100 Murcia, Spain; mtasoler@um.es; 4 Departamento de Morfología, Anatomía y Embriología, Facultad de Veterinaria, Universidad de Las Palmas de Gran Canaria, Trasmontaña, Arucas, 35416 Las Palmas de Gran Canaria, Spain; alberto.arencibia@ulpgc.es; 5 Departamento de Biología—CESAM, Campus Universitario de Santiago, Universidade de Aveiro, 3810-193 Aveiro, Portugal; a.lopez@ua.pt; 6 Coordinadora para el Estudio de los Mamíferos Marinos (CEMMA), Ap. 15, Gondomar, 36380 Pontevedra, Spain; 7 Servicio de Experimentación Animal, Área Científica-Técnica de Investigación, Edificio Centro de Experimentación e Investigación Biomédica (CEIB), Universidad de Murcia, 30100 Murcia, Spain; pablo_cov@yahoo.es (P.C.F.); ngc2@um.es (N.G.C.)

**Keywords:** striped dolphin (*Stenella coeruleoalba*), common dolphin (*Delphinus delphis*), pilot whale (*Globocephala melas*), fetal development, nose, nasal cavity, endoscopy, PET/SPECT/CT, MRI, sectional anatomy, dissection, 3D reconstruction, histology, ontogeny

## Abstract

**Simple Summary:**

The developmental anatomy of the dolphin head has been studied mostly in single fetuses and few works have been made using a wide range of specimens. In this study, fetal specimens are the main subjects, but newborn, juvenile and adult specimens were also used. Our study analyzes the external nose and nasal cavities during pre- and postnatal development. The nose and nasal cavities were studied using a high-resolution endoscopy to analyze changes in the mucosa of fetal specimens, newborns and juveniles. Magnetic Resonance Imaging (MRI) was also used in fetuses to locate and identify significant structures. Computed Tomography (CT) allowed us to understand the development of the facial bones and the nasal cavity. The histological samples were compared with a horse, a terrestrial mammal with a complex nasal anatomy. Dissections and anatomical sections in two spatial planes were compared with MRI and CT studies. Endoscopy of the external nose showed interesting morphological changes as only two different diverticula (air sacs) were observed in the vestibular part and one recess in the respiratory and olfactory part. We conclude that nasal cavity development of the striped and common dolphins and the pilot whale is simpler than in the bottlenose dolphin and the melon is part of the nose both anatomically and functionally.

**Abstract:**

Our goal was to analyze the main anatomical structures of the dolphin external nose and nasal cavity from fetal developmental stages to adult. Endoscopy was used to study the common development of the external nose and the melon, and nasal mucosa. Magnetic resonance imaging (MRI) and anatomical sections were correlated with anatomical sections. Computed tomography (CT) was used to generate 3D reconstructions of the nasal bones and nasal cavities to study its development. Dissections, histological and pathological studies were carried out on the nasal mucosa to understand its function. These results were compared with the horse. Endoscopy showed an external nose with two lips and the upper lip is divided by a groove due to the nasal septum and an obstruction of right nasal cavity was diagnosed in a newborn. Two diverticula (air sacs) were found in the nasal vestibule and an incisive recess (premaxillary sac) in the nasal cavity. These findings were corroborated by 3D reconstructions of the nasal cavities, MRI, anatomical sections and dissections. The presphenoid and ethmoid bones were fused at early stages of fetal development. The ethmoid is the last bone to ossify in the nasal cavity.

## 1. Introduction

Through evolution, mammals that colonized the aquatic environment have undergone numerous adaptations in their anatomical structures, especially those of the cephalic region [1]. These adaptative changes are mainly seen in the asymmetry and telescoping of the skull [2,3,4] and have direct implications in feeding [5], mechanical protection of cephalic structures, echolocation, breathing and diving. The repositioning of the bones of the dorsal skull affect the position of the nasal opening [6], whose dorsal position enables breathing while the animal is on the surface. Along with the air sacs and sinuses which have respiratory, vocal and structural functions [7,8,9,10], these modifications in cephalic anatomy results in the most highly evolved nose of all mammals.

There are not many articles in the scientific literature about the cetacean upper respiratory system, and some of the existing studies differ in their descriptions of structures and the terminology used to identify these structures... Most of those articles are about adult specimens, either odontocetes [11,12,13,14,15,16,17,18,19,20] or mysticetes [21,22,23,24] and many of them focused on sound production and biosonar [14,25,26]. The few papers on fetal specimens [27,28,29,30,31,32] do not cover different stages of development and only show one fetus without comparison with other fetal stages, essential to the understanding of cetacean ontogeny and its application in the fields of biology and veterinary medicine [29]. The development of the bones and other nasal structures continues post-partum with opening of the blowhole, expansion of the lungs and independent breathing. In the current study, we analyze the nasal complex of 22 odontocetes belonging to three species: (striped dolphin *Stenella coeruleoalba*, common dolphin *Delphinus delphis* and pilot whale *Globicephala melas*) of all ages (16 fetuses of different stages, three newborn, two juvenile and three adults). We applied several diagnostic techniques: computed tomography (CT), magnetic resonance imaging (MRI), anatomical sections, dissections, 3D reconstructions, histology and histopathology. Endoscopy is commonly used in dolphin medicine especially to view the lower respiratory tract (lungs and bronchus) [33,34,35,36,37] but is not often used to visualize the nasal passages, a region of key importance, not only in live animals, but also during necropsies to confirm any pathology incompatible with a correct air passage and therefore likely to interfere with vital activities such as diving and feeding that will decrease the life expectancy of the animal.

A comparison with terrestrial mammals will serve also to describe the structures of the head following the Illustrated Veterinary Anatomical Nomenclature [38] and to better understand the actual function and position of the different hard and soft tissue structures that form the nasal cavity of small cetaceans.

## 2. Materials and Methods

### 2.1. Animals

A total of sixteen prenatal, three perinatal specimens, two juvenile and four adult dolphins; one foal fetus and two adult horses were used in this study (Table 1). Additional information is located in Table S1 (Supplementary Materials). The mothers of each fetus were stranded along the Spanish Atlantic coast. The youngest newborn specimen was stranded on the Spanish African coast and the two others on the Mediterranean coast. The juvenile and adult specimens were stranded on the Spanish Mediterranean coast. All stranded dolphin specimens were found dead, two horse cadaver heads were obtained from the Orihuela abattoir; consequently, ethics committee clearance was not necessary. Endoscopic studies were made between September 2019 and January 2020 in a Veterinary Clinic (“Bonafé”), La Alberca (Murcia). Fourteen fetuses, one newborn and one adult specimen were transported to the image analysis units to perform scans. Six fetuses, one newborn and one adult were transported to the dissection room for silicone injections. The silicone was injected through the blowhole towards the nasal cavity, filling the vestibule first. The amount of silicone went from 1 to 8 cm^3^ in the fetuses, 16 cm^3^ in the newborn and 20–28 cm^3^ in the adult. After that, the blowhole was covered with a tight elastic band to avoid backflow of the silicone. Two adult specimens were used to obtain anatomical sections, one of them for histological analysis, one newborn for pathological study and two adults for dissection. One horse specimen was sectioned and the other dissected.

### 2.2. Endoscopy

A fixed endoscopy unit (Karl Storz Autocon 200, Tuttlingen, Germany) located at Clínica Veterinaria “Bonafé”, La Alberca (Murcia), Spain with a camera processor (Storz image 1 hub, camera head Karl Storz Image 1 H3 HD, a Storz power led 175) was used to obtain endoscopic images. For the external nose we employed a forward telescope (0° enlarged view, diameter 3 mm, length 14 cm, with incorporated fiber optic light transmission) and for the nasal cavity we used a forward-oblique telescope 30°, diameter 2.7 mm, length 18 cm, with incorporated fiber optic light transmission.

The endoscopic procedure was performed on the fetuses and juvenile specimens following standard protocols. Each specimen was placed in ventral recumbency and facing the endoscopist. Initial image acquisition was of the external nose morphology, using an optic of 4 mm diameter and 0° vision angle. Following this, the nasal cavity was examined by introducing an optic of 2.7 mm and 30° vision angle protected by a 3 mm sheath forming an irrigation channel, visualizing first the vestibule, then turning the optic to obtain a complete exposition of this part of the nasal cavity. After visualizing the nasal plugs, observation and study of the respiratory tract within the nasal cavity was conducted. For this purpose, and with the specimens in a lateral position, the endoscope was introduced through the vestibule towards this respiratory part, which allowed us to observe structures such as the incisive recess and the choanae, while irrigating physiological serum through the endoscopy irrigation channel to clean cavities and to obtain good images. All endoscopic images were stored in external and internal hard disks at the CVB (Bonafé Veterinary Clinic) and at the Department of Anatomy and Embryology, Facultad de Veterinaria, Universidad de Murcia, Spain.

### 2.3. Magnetic Resonance Imaging

Magnetic Resonance images were obtained with a high-field MR apparatus (General Electric Sigma Excite, Schenectady, NA, USA; Centro Veterinario de Diagnóstico por Imagen de Levante, Ciudad Quesada, Alicante, Spain), 1.5 Tesla using a human quad knee coil (dde1 to 3, 5, 7, 9 to 12, gma1), wrist coil (scop1, dde1) and head coil (dde13 and 14). All dolphin specimens were positioned in ventral recumbency. The MR images were transferred to a DICOM workstation. MR images were analyzed with Radiant DICOM viewer. MRI parameters used are in Table S2 (Supplementary Materials). All MR images were stored in external and internal hard disks at the CVDIL (Veterinary Center for Imaging Diagnosis), Ciudad Quesada, Alicante, Spain and at the Department of Anatomy and Embryology, Facultad de Veterinaria, Universidad de Murcia, Spain.

### 2.4. Computed Tomography and 3D Reconstruction of Bony Nasal Cavity

Four fetuses were scanned with Positron Emission Tomography (PET), Single Photon Emission Computed Tomography (SPECT)—Computed Tomography (CT) (PET/SPECT/CT AlbiraTM Systems, Valencia, Spain; Centro de Investigación Biomédica, Universidad de Murcia, Murcia, Spain). The following parameters were used: single-slice: 1 detector arrays; type of acquisition: helical; thickness: 0.125 mm; image reconstruction interval or index: 0.0125 mm; pitch: 0; tube rotation time: 0.12 s; mA: 0.4; Kv: 45; FOV: 68 cm; Matrix dimensions: 2240 × 2360; reconstruction algorithm: FBP filtered back projection; WW: 600/WL: 300). 

Three fetuses (one newborn and one adult) were scanned with a CT (General Electric Medical Systems-HiSpeed dual; Hospital Clínico Veterinario, Universidad de Murcia, Spain). All dolphin specimens were positioned in ventral recumbency. The following parameters were used: multislice: two detector arrays; type of acquisition: helical; thickness: 1 mm; image reconstruction interval or index: 2.5 mm; pitch: 0.35; tube rotation time: 1 s; mA: 45 (dde2), 50 (dde6), 55 (scog1, gma1), 60 (dde9,dde13), 75 (scomu1), 80 (dde14), 150 (scomu5); Kv: 120; image field of view: 40 cm; acquisition matrix: 512 × 512; reconstruction algorithm: standard; WW: 400/WL: 40) (Table 1).

All CT images were transferred to a DICOM workstation and CT images were analyzed with AMIRA for Fei Systems 5.6 (Thermo Fisher Sci and Zuse Institute, Berlin, Germany). Volume rendering was generated to obtain 3D renderings of internal anatomy. All CT images were stored in external and internal hard disks at CEIB (Experimental Center of Biomedical Research) building, SAI (Support Research Facility) building and at the Department of Anatomy and Embryology, Facultad de Veterinaria, Universidad de Murcia, Spain.

### 2.5. Computed Tomography and 3D Reconstruction of Nasal Cavity Spaces

The nasal cavities of several fetal, newborn and adult specimens were injected with Silicone Xiameter ^©^ RTV-4230-E Base, 2 to 30 mL depending on size of specimen, (Dow Corning Co, Midland, MI, USA; Dissection room, Facultad de Veterinaria, Murcia, Spain) to obtain nasal endocasts and to enhance CT contrast properties. Injected specimens were scanned with CT and DICOM images were used to obtain 3D reconstructions of the nasal cavities. Three-dimensional reconstructions of the nasal cavity were obtained using AMIRA for Fei Systems 5.6. All Dicom images were stored in external and internal hard disks at SAI (Support Research Facility) building and at the Department of Anatomy and Embryology, Facultad de Veterinaria, Universidad de Murcia, Spain.

### 2.6. Anatomic Evaluation: Sectional Anatomy and Dissection Techniques

One newborn and one juvenile striped dolphin (*Stenella coeruleoalba*) were frozen at −20 °C prior to obtaining coronal and sagittal sections of the head. One adult striped dolphin (*Stenella coeruleoalba*) was frozen at −46 °C prior to obtaining sagittal sections. All specimens were cut with a band saw (Anatomical Lab., Department of Anatomy and Embryology, Universidad de Murcia, Murcia, Spain), obtaining 0.5–0.7 cm thick slices. Head sections and slices were immersed in 10% formaldehyde for preservation and then stored in a cooling chamber (5 °C) at the Department of Anatomy and Embryology, Facultad de Veterinaria, Universidad de Murcia, Spain.

Fourteen fetuses were preserved by immersion in formaldehyde (10%) and two fetuses were fixed with embalming solution injected into the umbilical arteries and veins. In a striped dolphin (*Stenella coeruleoalba*) (scomu5), the external jugular vein and the left and right atria were injected with embalming solution using an electrical pump. After 48 h, the arteries and veins were injected with red and blue latex, respectively. A deep head dissection of one ill newborn (scoce1) was made to observe abnormal anatomy of the vestibule and nasal cavity and an adult specimen was dissected to observe the normal anatomy of nasal cavity (Table 1). All specimens used were stored in a cooling and freezer chambers at the Department of Anatomy and Embryology, Facultad de Veterinaria, Universidad de Murcia, Spain.

### 2.7. Histological Analysis

The mucosa of the nasal cavity (vestibule and respiratory and olfactory parts) was histologically analyzed in one adult dolphin specimen. Elongated rectangles of nasal mucosa were removed at different levels of the nasal cavity. Samples were oriented perpendicular to the paraffin block base and then processed using a special saw. Paraffin blocs were cut to obtain slices. Routine histological processing was carried out and sections were stained with Haemotoxylin and Eosin. Samples were then photographed with a computed light microscope (Axioskop 40, Zeiss, Jena, Germany) with an incorporated Insight 2 Axiocam 105 color camera. Histological sections were stored at the Department of Anatomy and Embryology, Facultad de Veterinaria, Universidad de Murcia, Spain.

## 3. Results

### 3.1. Endoscopic Study of the External Nose and Nasal Cavity

The endoscopic figures are described by columns, observing in the left column the external nose closed in its natural position to avoid the entrance of amniotic liquid into the lungs.

#### 3.1.1. External Nose

**Figure 1:**
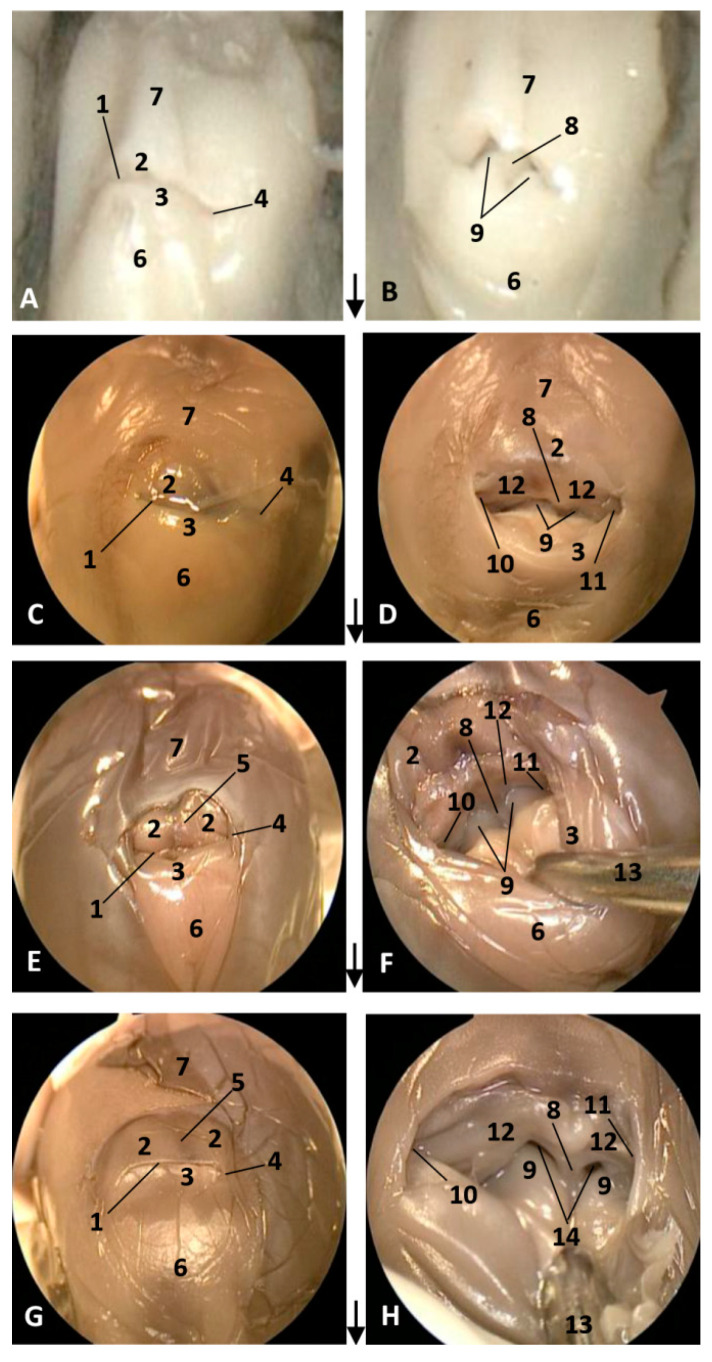
Endoscopic images of the external nose and nasal cavity at the level of nasal vestibule. Images are oriented so that the left side of the head is to the right of the image and rostral is at the bottom (**arrow**). (**A**,**B**) 1.5 months, dde1; (**C**,**D**) 3.5 months, dde2; (**E**,**F**) 4 months, dde3; (**G**,**H**) 4.5 months, scop1. 1, Rima naris; 2, Upper lip; 3, Lower lip; 4, Angulus naris; 5, Nasal groove; 6, Melon; 7, Forehead; 8, Nasal septum: membranous part; 9, Nasal plugs; 10. Vestibule: right diverticulum (vestibular sac); 11, Vestibule: left diverticulum (vestibular sac); 12, Vestibular folds; 13, Delicate hook; 14, Nasal cavity: respiratory part.

In the youngest fetus (dde1), a common dolphin *Delphinus delphis*, a very small protuberance was observed between the forehead and the melon. It was seen between a semi-circular line (U-shaped) (rima naris), slightly concave right to angulus naris, closing it hermetically. We observed two lips, the upper (caudal) close to the forehead and the lower (rostral) close to the primordial melon. The lower lip was a little bit prominent compared to the upper (Figure 1A). In the next fetus (dde2), a common dolphin (*Delphinus delphis*), both lips are prominent, the upper due to the fact that it underlies the vestibular fold, muscle fibers and the nasal bones mesenchyme and the lower lip, due to the proximity to the developing melon. The lips line (rima) could be now observed as slightly convex (Figure 1C). In the third common dolphin (*Delphinus delphis*) fetus (dde3) (4.5 months gestation) the epidermis of the external nose differs from the forehead epidermis, with a different colour (light pink) and with a clear separation edge of the upper lip and the forehead skin. This lip, now prominent, was clearly divided vertically by a supranasal groove. The lower lip was undivided and elevated right to the contact with the melon, which has a similar tone to that of the nose. The rima naris was slightly convex (Figure 1E). In the next fetus (scop1), a striped dolphin (*Stenella coeruleoalba*), the external nose maintained its change in colour with respect to adjacent areas. The rima naris shows a slight concavity in both angulus. The upper lip is prominent, compared to the common dolphin (*Delphinus delphis*) fetuses with a light groove dividing them in two while the lower lip descends rostrally, forming a valley, extending rostrally to the melon prominence (Figure 1G).

**Figure 2:**
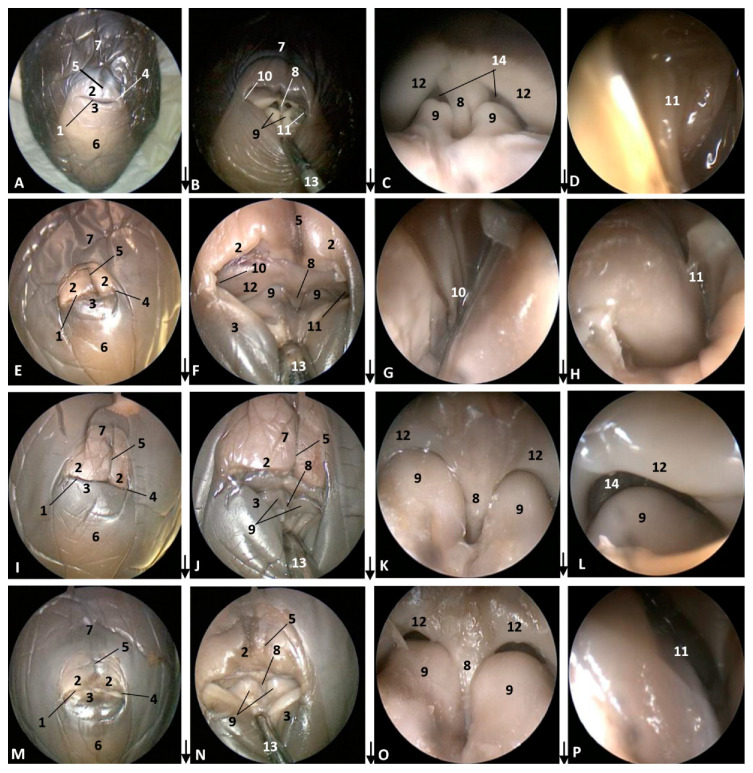
Endoscopic images of the external nose and nasal cavity at level of the vestibule. Images are oriented so that the left side of the head is to the right of the image and rostral is at the bottom (**arrow**). Detail of monkey or phonic lips from now on (**K**,**L**,**O**). (**A**–**D**) 5 months, gma1; (**E**–**H**) 5.5 months, dde4; (**I**–**L**) 6 months, dde8; (**M**–**P**) 7 months, dde9. 1, Rima naris; 2, Upper lip; 3, Lower lip; 4, Angulus naris; 5, Nasal groove; 6, Melon; 7, Forehead; 8, Nasal septum: membranous part; 9, Nasal plugs; 10. Vestibule: right diverticulum; 11, Vestibule: left diverticulum; 12, Vestibular folds; 13, Delicate hook; 14, Nasal cavity: respiratory part.

In the pilot whale (*Globocephala melas*) fetus (gma1), the external nose presented a whitish pigmentation extending to the melon, while the pigmentation of the rest of the head is dark grey. The rima naris is slightly convex and differs from the prominent upper lip divided by the supranasal groove. The rostral lip is visible (Figure 2A). The three next fetuses of the common dolphin (*Delphinus delphis*), more developed than the pilot whale (*Globocephala melas*) fetus, showed the same features as the common dolphin (*Delphinus delphis*) fetus (dde3), with a separation area holding the nose and melon, though the depigmentation can only be observed at the upper lip. The convexity in the rima naris is very slight (Figure 2E,I,M).

**Figure 3:**
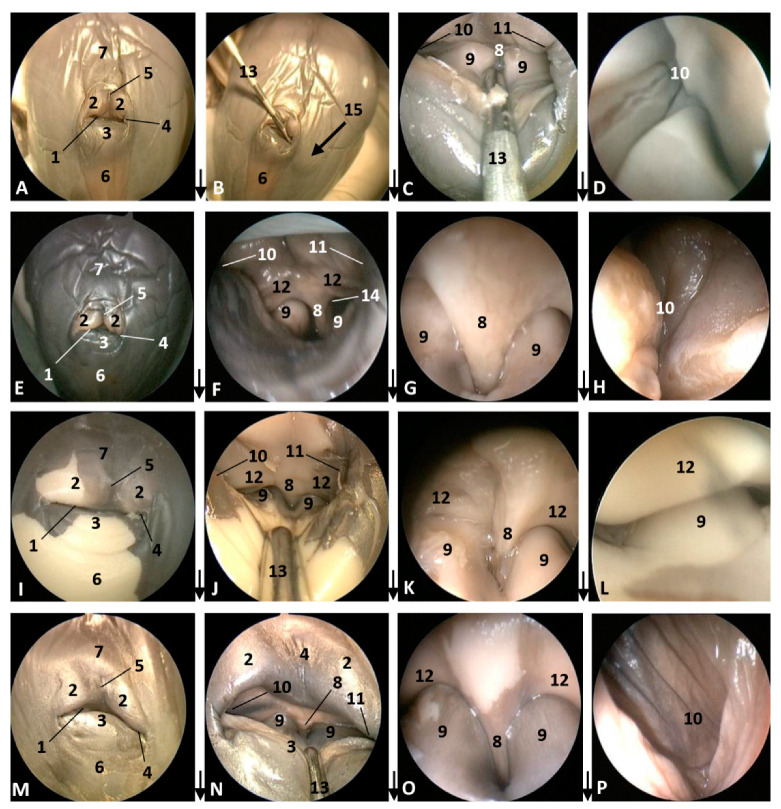
Endoscopic images of the external nose and nasal cavity at the level of vestibule. Images are oriented so that the left side of the head is to the right of the image and rostral is at the bottom (**arrow**). (**A**–**D**) 7.5 months, dde10; (**E**–**H**) 8 months, dde11; (**I**–**L**) 8.5 months, dde12; (**M**–**P**) 9 months, dde13 1, Rima naris; 2, Upper lip; 3, Lower lip; 4, Angulus naris; 5, Nasal groove; 6, Melon; 7, Forehead; 8, Nasal septum: membranous part; 9, Nasal plugs; 10. Vestibule: right diverticulum; 11, Vestibule: left diverticulum; 12, Vestibular folds; 13, Delicate hook; 14, Nasal cavity: respiratory part; 15, Diverticulum extension.

The four next common dolphin (*Delphinus delphis*) fetuses, from 7 to 9 months of gestation, mantain the external features described in the last three, though the midline rima naris forms a triangle with respect to the supranasal groove, and it appears as a horizontal line (Figure 3A,E,I). In the last fetus a light concavity is seen (Figure 3M).

**Figure 4:**
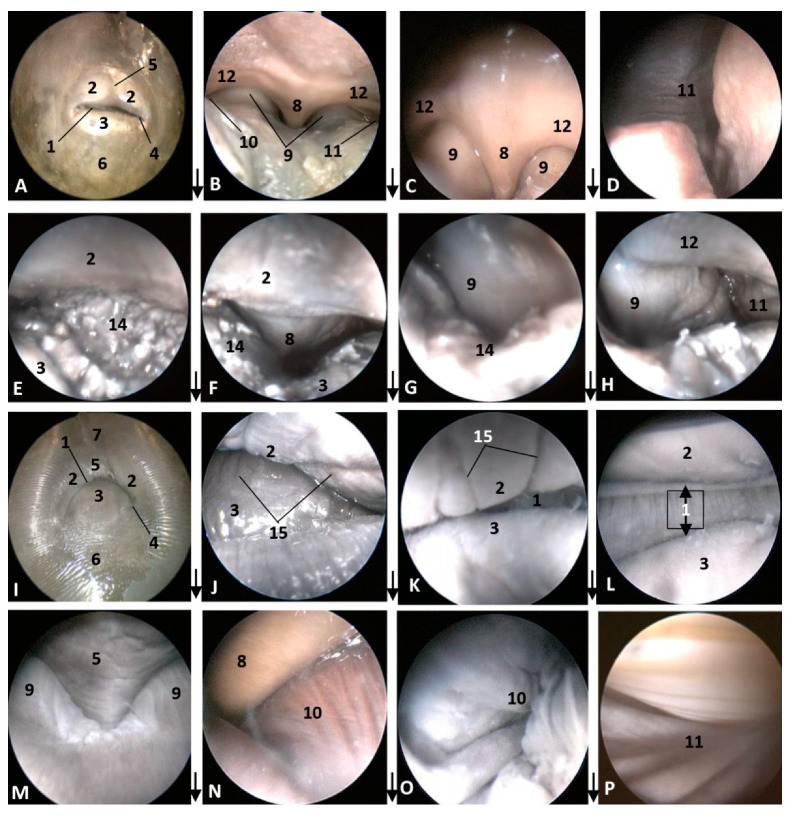
Endoscopic images of the external nose and nasal cavity at the level of vestibule. Images are oriented so that the left side of the head is to the right of the image and rostral is at the bottom (**arrow**). (**A**–**D**) 10 months, dde14; (**E**–**H**) newborn, scoce1; (**I**–**P**) juvenile, scomu4. 1, Rima naris; 2, Upper lip; 3, Lower lip; 4, Angulus naris; 5, Nasal groove; 6, Melon; 7, Forehead; 8, Nasal septum: membranous part; 9, Nasal plugs; 10. Vestibule: right diverticulum; 11, Vestibule: left diverticulum; 12, Vestibular folds; 13, Delicate hook, 14. Hyperkeratosis; 15, Striation marks.

In the last common dolphin (*Delphinus delphis*) fetus, the depigmentation of the nose and upper lip remains and misses the delimitation of the nose and melon with respect to the rest of the head. The rima naris keeps the concavity towards the angulus naris, commissures or nose angles (Figure 4A). In the next specimen, a newborn striped dolphin (*Stenella coeruleoalba*), showed a hyperkeratosis between the two lips and the right part of upper lip preventing the access of the endoscope (Figure 4E). In the last specimen studied, a juvenile striped dolphin (*Stenella coeruleoalba*), the nose was tightly closed and uniform pigmentation was seen. The upper lip is divided in two by the supranasal groove. The rima naris was curved towards the commissures or angulus naris and presented many folds sinking to the rima. The lower lip is undivided and prominent remaining close to the melon (Figure 4I). Between both nasal lips, there are striations and a double internal fold hermetically sealing the lips (Figure 4J,K,L).

#### 3.1.2. Nasal Cavity: Vestibule

In the second, third and fourth columns the external nose was opened allowing us to see the first part of the nasal cavity, the vestibule (Figure 1, Figure 2, Figure 3 and Figure 4).

After caudal retraction of the skin of the forehead region of the least developed common dolphin (*Delphinus delphis*) fetus (dde1), the nose was opened to expose the vestibule. Only the membranous part of the nasal septum was seen as were the two nasal plugs. The mucosa is of a similar colour of that of the epidermis, a little clearer at the plugs (Figure 1B). In the next specimen (dde2), we could also see the vestibular folds dorsal to the nasal plugs and laterally the entry to the nasal diverticula (Figure 1D). In the third common dolphin (*Delphinus delphis*) fetus (dde3), the lower lip was pulled rostrally in order to observe the vestibule. The nasal plugs could be seen between the nasal septum. The vestibular folds along with the membranous part of the nasal septum stick to the plugs, closing the meatus tightly. The nasal diverticula (vestibular sacs) (Figure 1F) of the striped dolphin (*Stenella coeruleoalba*) fetus (scop1), already more developed, showed a mucosa with a whitish tone which was uniform throughout the entire vestibule. Also, the meatus was opened between the ‘monkey lips’ (both the nasal plugs and the vestibular folds) (Figure 1H). In the pilot whale (*Globocephala melas*) fetus (gma1), the mucosal colour of the vestibule remains the same (Figure 2B,C), though it differs from the greyish tone inside the lips (Figure 2B). The mucosa of the nasal diverticulum remained dark and had folds (Figure 2D). In the three next common dolphin (*Delphinus delphis*) fetuses, the vestibular mucosa goes from pink, to grey to white (Figure 2F,J,N), shown in detail (Figure 2K,L,O). The mucosa of the diverticula has greyish folds arranged in a dorsoventral direction (Figure 2G,H,P). The next group of common dolphin (*Delphinus delphis*) fetuses had a more advanced gestation time. A clamp was used to mark the internal extension of one diverticulum (Figure 3B). We subsequently observed prominent nasal plugs, the nasal septum and well-defined folds and a greyish mucosa, different from the earlier fetal periods (Figure 3C,F,J,N,G,L,O and Figure 4B,C). The mucosa of both left and right diverticula was growing in anfractuosity and the longitudinal folds more abundant (Figure 3D,H,P and Figure 4D).

The newborn (scoce1) was diagnosed with epithelial inflammation with local hyperkeratosis probably caused by a congenital alteration. This obstructed the right nasal cavity (Figure 4E). This specimen had a thickened membranous part of the nasal septum so the nasal plugs are barely visible (Figure 4F). Only the left nasal plug and the dilated mucosa of left diverticulum were visible (Figure 4G,H).

In the juvenile striped dolphin (*Stenella coeruleoalba*), the mucosa of vestibule was wrinkled with striations and was tightly adhered to the plug, septum and vestibular folds (Figure 4M,N). The mucosa of the nasal diverticula presented numerous longitudinal folds (Figure 4O,P).

#### 3.1.3. Nasal Cavity: Respiratory and Olfactory Part

In this part of the nasal cavity, nasal cavities (left and right) were analyzed from superficial to deep regions after passing the nasal plugs. Each row corresponds with each studied specimen, with the first row corresponding to the specimen with the earliest fetal development. This part of the study started analyzing more developed specimens in which the nasal cavity diameter allowed easy passage of the endoscope. In the nasal cavity, we could distinguish four walls: the caudal, once the endoscope passed the nasal plug; the rostral wall, ventral to the nasal plug and containing the incisive recess (premaxillary sac). The lateral wall is the external wall of both cavities and finally, the median wall, built by the vomer bone, separating both choanae.

**Figure 5:**
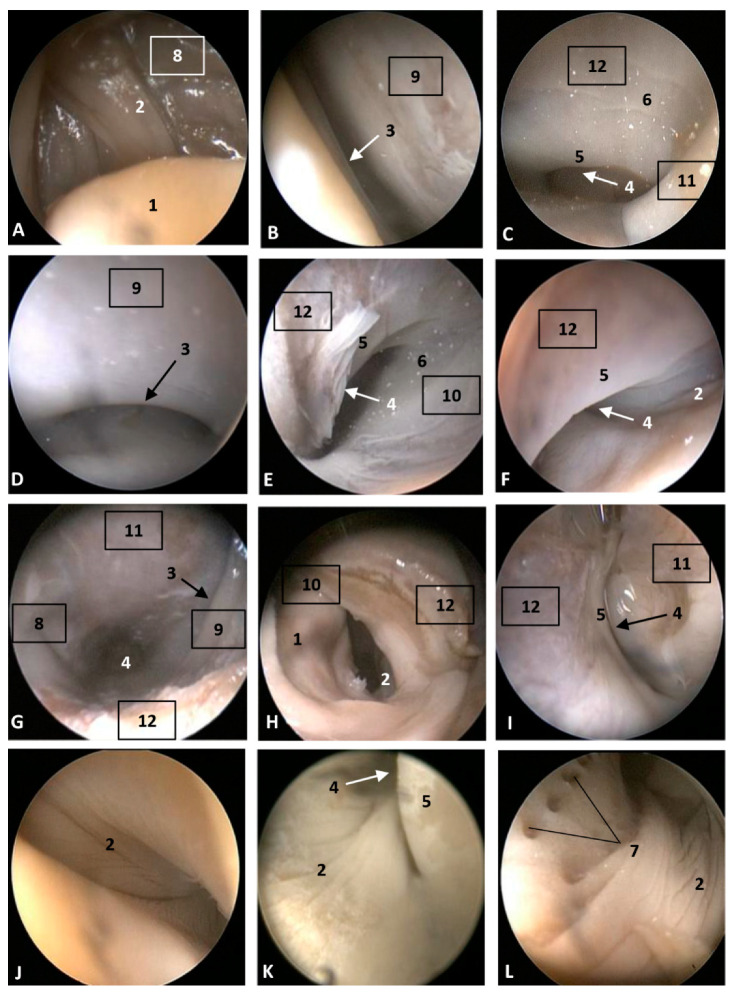
Endoscopic images of the nasal cavity at the level of respiratory and olfactory parts. Arrows show the entry to the incisive recess (3) and to the nasopharynx (4) (**A**,**B**,**H**) Right nasal cavity, (**C**–**G**,**I**–**L**) Left nasal cavity, (**A**–**C**) 5.5 months, dde4; (**D**–**F**) 6 months, dde7. (**G**–**I**) 7 months, dde9; (**J**–**L**) Detailed images. 8.5 months, dde12. Walls indicate orientation. Vertical view. 1. Nasal plug; 2, Longitudinal folds; 3, Incisive recess (premaxillary sac); 4, Choanae; 5, Nasal septum: bony part; 6, Small vesicles; 7, Small fossae; 8, Caudal wall; 9, Rostral wall; 10, Right lateral wall; 11, Left lateral wall; 12, Medial wall.

The study starts in a common dolphin (*Delphinus delphis*) fetus of approximately 5.5 months of gestation. We had observed, caudal to the nasal plug, how the mucosa of longitudinal folds was placed vertically towards the floor of the right nasal cavity (Figure 5A). On turning the endoscope rostrally, we observed the incisive recess (Figure 5B). The mucosa of the deepest part of the recess in the left nasal cavity contained small vesicles. Also, at the bottom of the nasal cavity we could see the choanae and the bony portion of the nasal septum (Figure 5C). Unlike the vestibule, the mucosa of the respiratory and olfactory part shows a pinkish colour in every specimen studied. The presence of vesicles in the mucosa and longitudinal folds was clearly seen (Figure 5D–L). Small mucosal fossae close to the choanae were observed mainly in the common dolphin (*Delphinus delphis*) fetus 8.5 months of gestation (Figure 5L).

**Figure 6:**
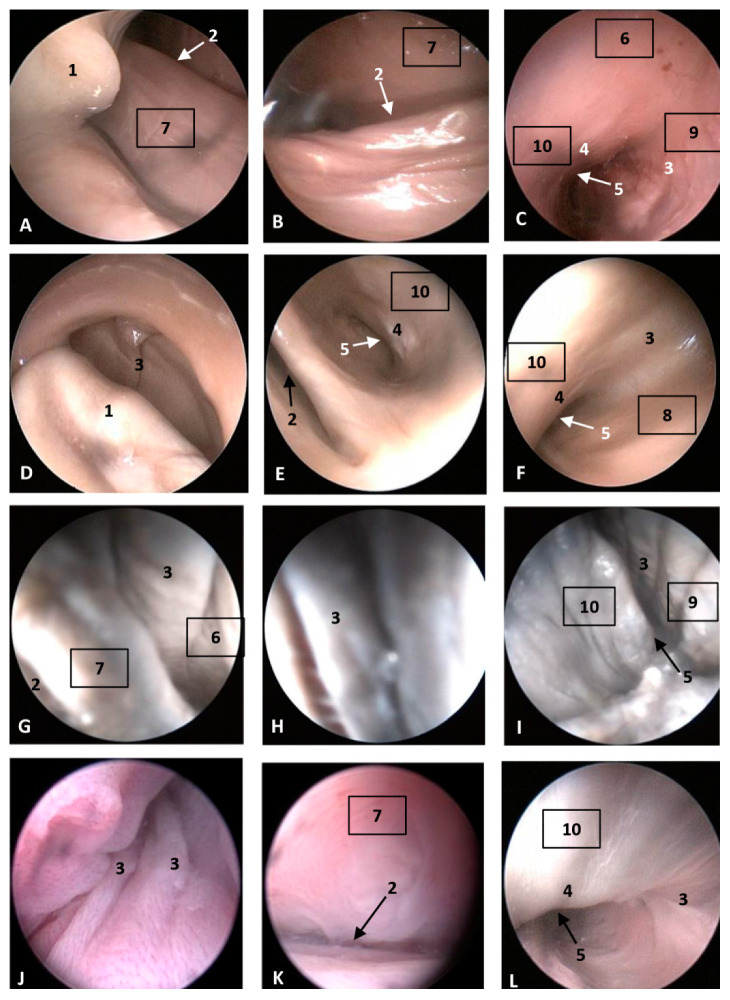
Endoscopic images of the nasal cavity at the level of respiratory and olfactory parts. Arrows show the entry to the incisive recess (2) and to the nasopharynx (5) 9 months, dde13. (**A**–**C**,**L**) Left nasal cavity. (**D**–**K**) Right nasal cavity. (**A**–**C**) 9 months, dde13; (**D**–**F**) 10 months, dde14; (**G**–**I**) Hypertrophied longitudinal folds. Newborn, scoce1; (**J**–**L**) juvenile, scomu4. Walls indicate orientation. Vertical view. 1, Nasal plug; 2, Incisive recess; 3, Nasal folds; 4, Nasal septum: bony part. 5, Choanae; 6, Caudal wall; 7, Rostral wall; 8, Right lateral wall; 9, Left lateral wall; 10, Median wall.

In the last two common dolphin (*Delphinus delphis*) fetuses closest to birth, we highlight that the incisive recess is more visible and widened and the mucosa had few folds, a flat aspect and a pinkish colour (Figure 6A–F). In the newborn (scoce1), we could only study the left respiratory part of the nasal cavity since the right one was blocked and was impossible to introduce the endoscope due to hyperkeratosis. The mucosa was greyish in colour and there was inflammation of the nasal mucosa folds. The incisive recess could be observed (Figure 6G–I).

In the last specimen studied with the endoscope, the striped juvenile dolphin, we observed the pinkish mucosa and thick folds, the incisive recess and the choanae (Figure 6J–L). Also, small fossae could be seen close to the choanae (Figure 6L).

### 3.2. MRI Study of the External Nose and Nasal Cavity

MRI were first used in the sagittal plane in order to have an overall vision of the whole nasal cavity and afterwards in three coronal planes at the level of vestibule, the incisive recess and finally at the choanal region.

**Figure 7:**
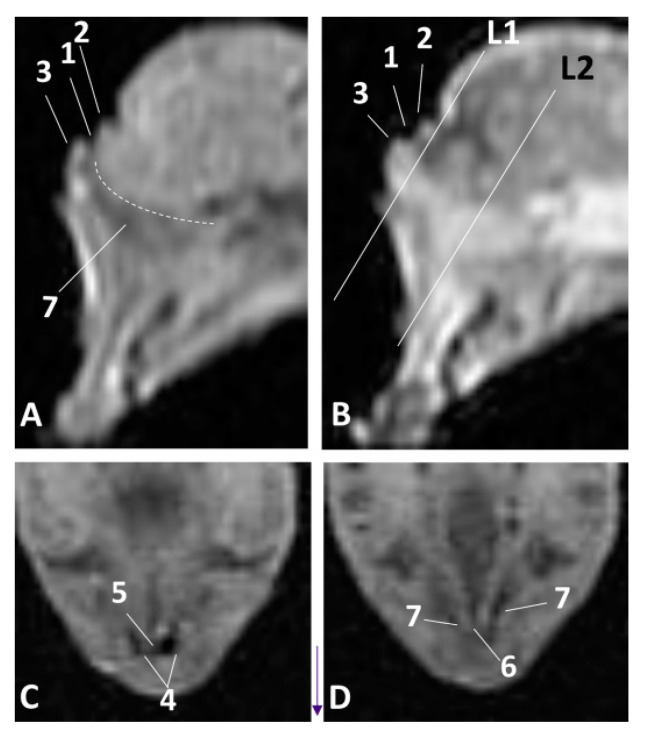
Magnetic Resonance Imaging (MRI) of the external nose and nasal cavity. MR sagittal images (**A**,**B**) are oriented so that the rostral is to the left and the caudal to the top. MR coronal images (**C**,**D**) are oriented so that the rostral is to the bottom and the caudal to the top. (**A**) T1-weighted Spin echo (SE) sagittal plane, (**D**) T2-weighted Fast Recovery Fast Spin Echo (FrFSE) sagittal plane. (**C**,**D**) T1-weighted SE coronal plane. (**C**) Level 1. (**D**) Level 2. 1.5 months, dde1. 1, Rima naris; 2, Upper lip; 3, Lower lip; 4, Nasal cavity: vestibule; 5, Nasal septum: membranous part; 6, Nasal septum: bony part; 7. Nasal cavity: respiratory part.

The least developed specimen of common dolphin (*Delphinus delphis*) dde1, corresponds to first stage of fetal development. The sagittal MRI allowed us to identify the external nose with two lips and the rima naris. The melon is beginning to develop and was observed hyperintense in both sagittal images. The nasal cavity from the vestibule to the choanae is shown as a mass of mesenchymal tissue slightly hypointense in T1 and hyperintense in T2 (Figure 7A,B). The coronal MRI at the vestibule level allows us to identify the nasal septum slightly hypointense at level of the vestibule (L1) and hyperintense at the respiratory part (L2). Nasal cavities were observed always hypointense (Figure 7C,D).

**Figure 8:**
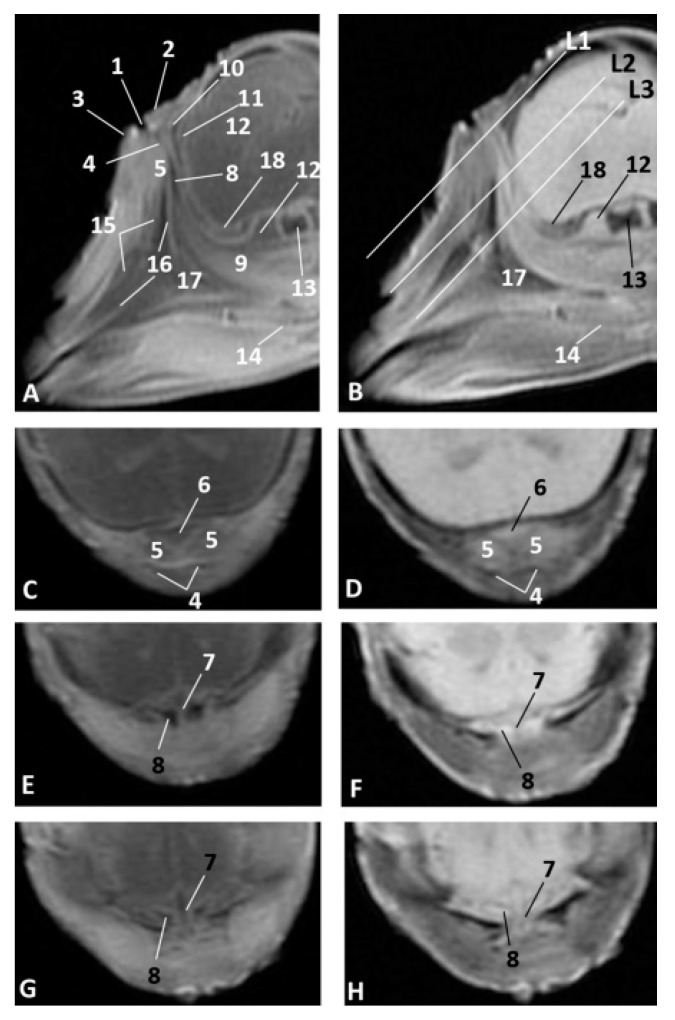
MRI of the external nose and nasal cavity. MR sagittal images (**A**,**B**) are oriented so that the rostral is to the left and the dorsal to the top. MR coronal images (**C**,**H**) are oriented so that the rostral is to the bottom and the caudal to the top. (**A**) T1-weighted SE sagittal plane, (**B**) T2-weighted FrFSE sagittal plane. (**C**,**E**,**G**) T1-weighted SE coronal plane (**D**,**F**,**H**) T2-weighted FrFSe coronal plane. (**C**,**D**) Level 1, (**E**,**F**) Level 2, (**G**,**H**) Level 3. 4 months, dde3. 1. Rima naris; 2, Upper lip; 3, Lower lip; 4, Nasal cavity: vestibule (left and right diverticula); 5, Nasal plugs; 6, Nasal septum: membranous part; 7, Nasal septum: bony part; 8, Nasal cavity: respiratory part; 9, Choanae: 10, Nasal bone; 11, Frontal bone; 12, Presphenoid bone; 13, Basisphenoid bone; 14, Pterygoid bone; 15, Incisive bone (premaxilla); 16, Maxillary bone; 17, Vomer bone; 18, Ethmoidal mesenchyme.

In the fetus of four months’ gestation, dde3, the sagittal sections clearly showed the external nose. The nasal cavity vestibule section does not show the developing cavities, although slightly hypointense (diverticula) were noted. The nasal plug could be seen as slightly hyper/hypointense in T1 and T2, and the respiratory part of the nasal cavity was hypointense in T1 and T2. The main bones which form the walls are more distinct within the mesenchyme especially the presphenoid, but the ethmoid bone was not clearly identified (Figure 2A,B). In the coronal sections we could see the nasal plugs hypointense in T1 and slightly hyperintense in T2. The nasal septum was observed with different intensity in T1 and T2 as well as the vestibule and respiratory parts of the nasal cavity (Figure 8C,H).

**Figure 9:**
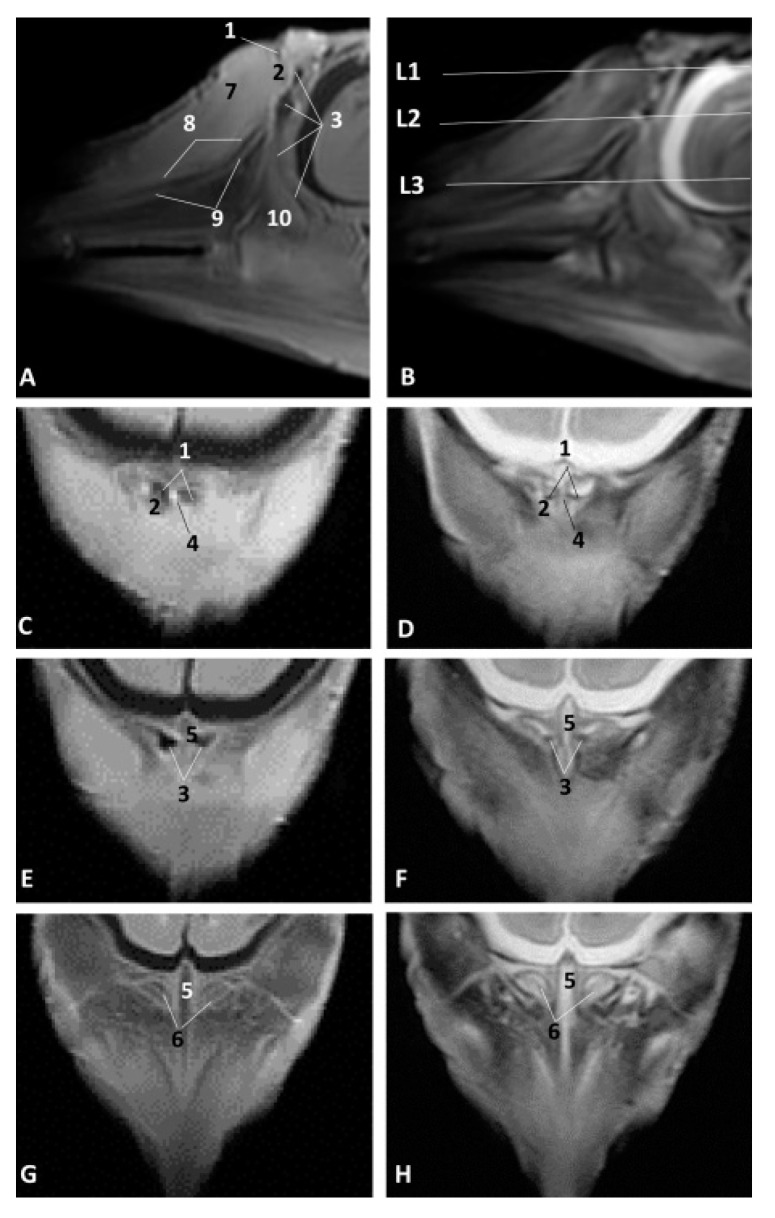
Images of the external nose and nasal cavity. MR sagittal images (**A**,**B**) are oriented so that rostral is to the left and dorsal to the top. MRI coronal images (**C**–**H**) are oriented so that the rostral is to the bottom and the caudal to the top. (**A**) T1-weighted SE sagittal plane. (**B**) T2-weighted FrFSE sagittal plane. (**C**,**E**,**G**) T1-weighted SE coronal plane, (**D**,**F**,**H**) T2-weighted FrFSE coronal plane. (**C**,**D**) Level 1. (**E**,**F**) Level 2. (**G**,**H**) Level 3. 4.5 months, scop1. 1, Nasal cavity: vestibule; 2, Nasal plug; 3, Nasal cavity: respiratory part; 4, Nasal septum: membranous part; 5, Nasal septum: bony part; 6, Choanae; 7, Melon; 8, Incisive bone; 9, Maxillary bone; 10, Vomer bone.

In a similar development stage (striped dolphin *Stenella coeruleoalba* fetus, scop1), we could better identify the lumen of the respiratory part of the nasal cavity, but the bones were less defined (Figure 9A–H).

**Figure 10:**
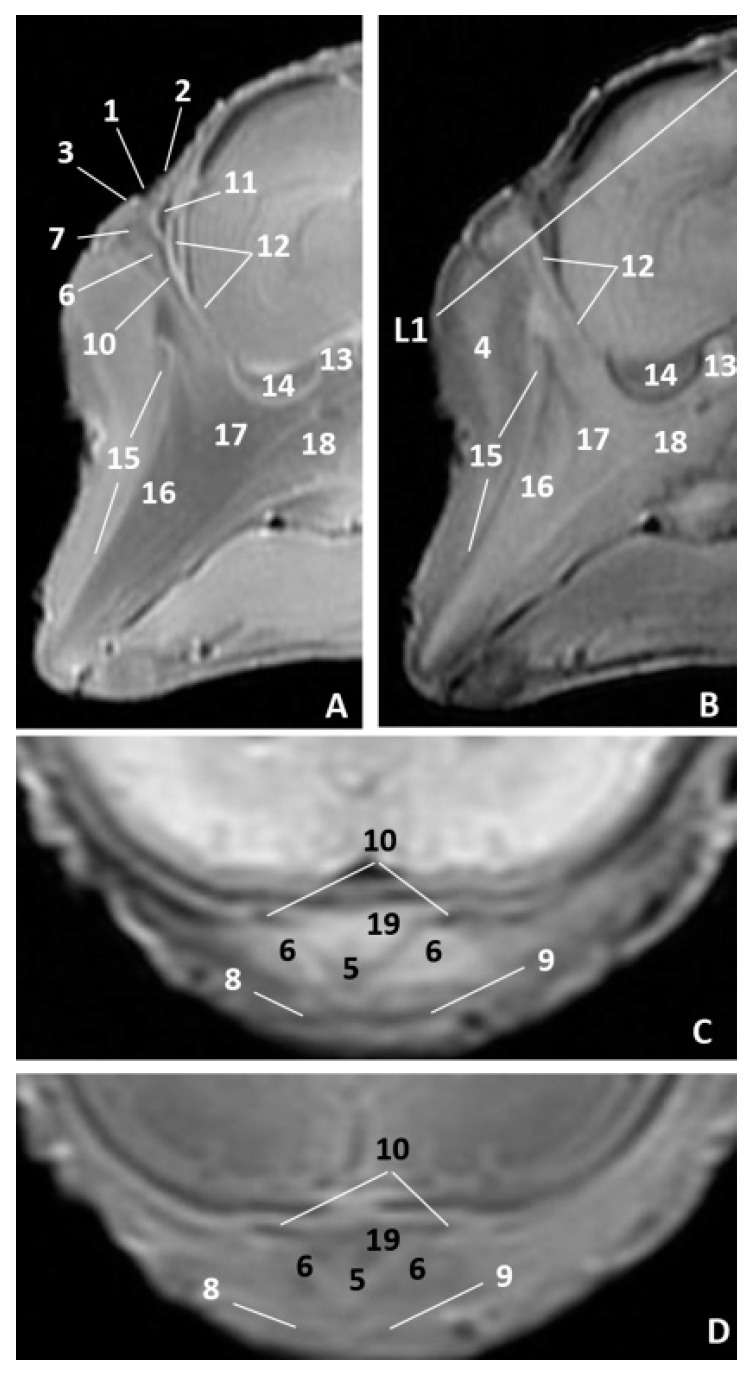
Images of the external nose and nasal cavity. MR sagittal images (**A**,**B**) are oriented so that the rostral is to the left and the dorsal to the top. MR coronal images (**C**,**D**) are oriented so that rostral is to the bottom and caudal to the top. (**A**) T1-weighted SE sagittal plane, (**B**) T2-weighted FrFSE sagittal plane. (**C**) T1-weighted SE coronal plane. (**D**) T2-weighted FrFSE coronal plane. (**C**,**D**) Level 1. 5 months, gma1. 1, Rima naris; 2, Upper lip; 3, Lower lip; 4, Melon; 5, Nasal septum: membranous part; 6, Nasal plugs; 7, Nasal cavity: vestibule; 8, Vestibule: right diverticulum; 9, Vestibule: left diverticulum; 10, Nasal cavity: respiratory part; 11, Nasal bone; 12, Frontal bone; 13, Presphenoid bone; 14, Ethmoid bone: perpendicular lamina; 15, Incisive bone; 16, Maxillary bone; 17, Vomer bone, 18, Pterygoid bone; 19, Vestibular folds.

In the pilot whale (*Globocephala melas*) fetus, gma1, we found better definition of the nasal plugs, the vestibule and respiratory parts of the nasal cavity in the coronal sections and most of the bony nasal cavity. At this stage, the perpendicular lamina of the ethmoid bone was seen clearly using MRI (Figure 10A–D).

**Figure 11:**
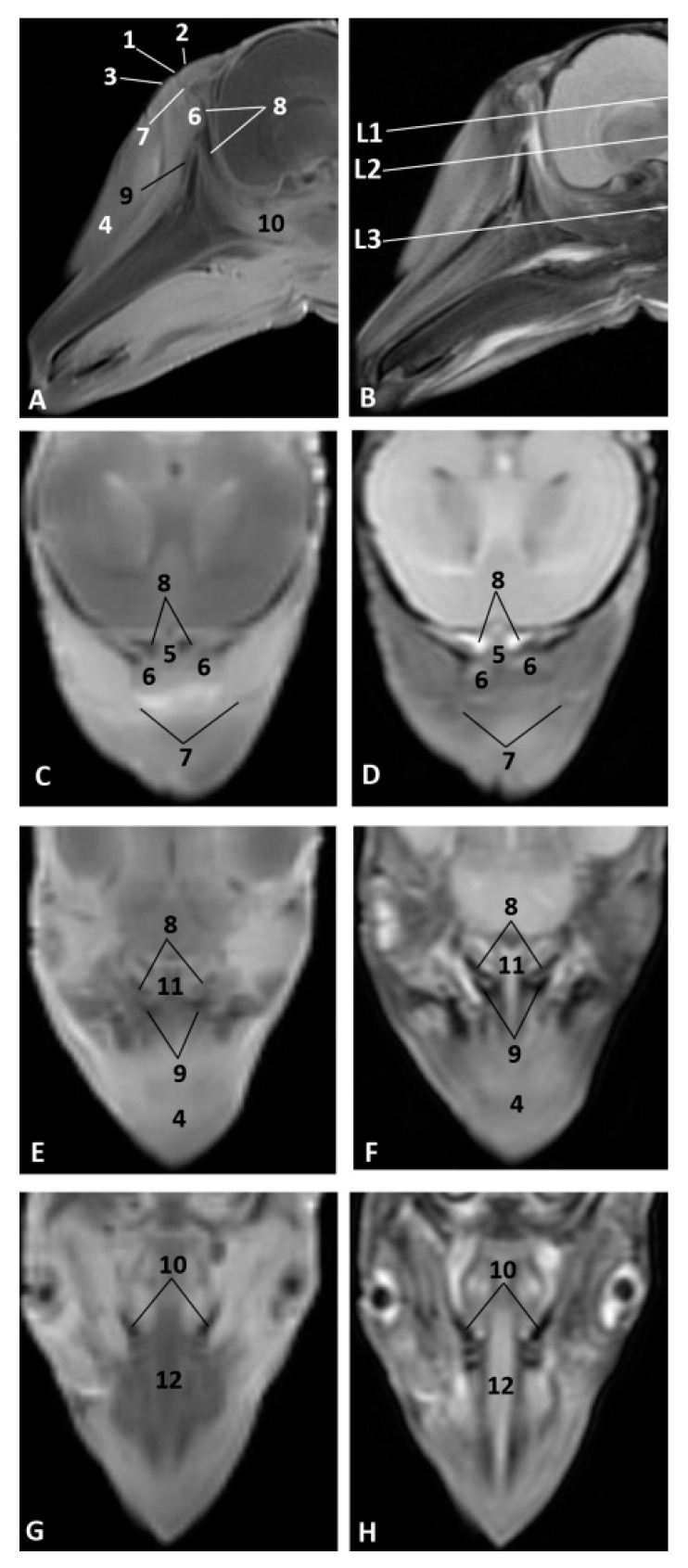
Images of the external nose and nasal cavity. MR sagittal images (**A**,**B**) are oriented so that the rostral is to the left and the dorsal to the top. MR coronal images (**C**,**H**) are oriented so that the rostral is to the bottom and caudal to the top. (**A**) T1-weighted SE sagittal plane, (**B**) T2-weighted FrFSE sagittal plane. (**C**,**E**,**G**) T1-weighted SE coronal plane. (**D**,**F**,**H**) T2-weighted FrFSE coronal plane. (**C**,**D**) Level 1. (**E**,**F**) Level 2. (**G**,**H**) Level 3. 6 months, dde7. 1, Rima naris; 2, Upper lip; 3, Lower lip; 4, Melon; 5, Nasal septum: membranous part; 6, Nasal plugs; 7, Nasal cavity: vestibule; 8, Nasal cavity: respiratory part; 9, Nasal cavity: incisive recess; 10, Choanae; 11, Nasal septum: bony part; 12, Mesorostral cartilage.

In the common dolphin (*Delphinus delphis*) fetus of six months’ gestation, dde7, the melon was observed as more developed and we identified the incisive recess using MRI, as well as the respiratory part of the nasal cavity, as a hypointense cavity in T1 and hyperintense in T2 (Figure 11A,B). In the three anatomical sections we also identified the nasal plugs, the nasal septum (membranous and bony parts) and the mesorostral cartilage (Figure 11C–H).

**Figure 12:**
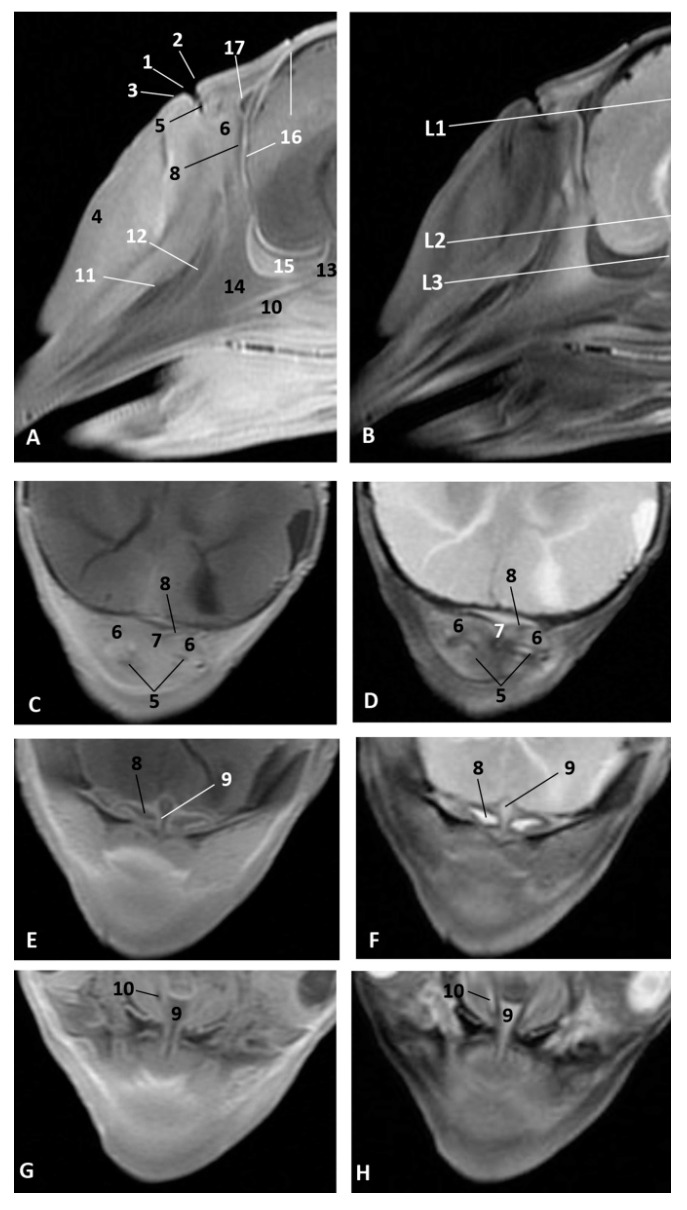
Images of the external nose and nasal cavity. MR sagittal images (**A**,**B**) are oriented so that the rostral is to the left and the dorsal to the top. MR coronal images (**C**–**H**) are oriented so that the rostral is to the bottom and caudal to the top. (**A**) T1-weighted SE sagittal plane, (**B**) T2-weighted FrFSE sagittal plane. (**C**,**E**,**G**) T1-weighted SE coronal plane. (**D**,**F**,**H**) T2-weighted FrFSE coronal plane. (**C**,**D**) Level 1. (**E**,**F**) Level 2. (**G**,**H**) Level 3. 7.5 months, dde10. 1, Rima naris; 2, Upper lip; 3, Lower lip; 4, Melon; 5, Nasal cavity: vestibule; 6, Nasal plugs; 7, Nasal septum: membranous part; 8, Nasal cavity: respiratory part; 9, Nasal septum: bony part; 10, Choanae; 11, Incisive bone; 12, Maxillary bone; 13, Presphenoid bone; 14. Vomer; 15, Ethmoid bone: perpendicular lamina; 16, Frontal bone; 17, Nasal bone.

In an advanced stage of fetal development all the anatomical structures of the nasal cavity could be seen perfectly defined (Figure 12A–H). We identified the perpendicular lamina of the ethmoid bone in the common dolphin (*Delphinus delphis*) fetus of 7.5 month’s gestation (dde10) as hyperintense in T1 and hypointense in T2. Between the frontal and ethmoid bones mesenchyme still is observed (not bony contact) (Figure 12A,B).

**Figure 13:**
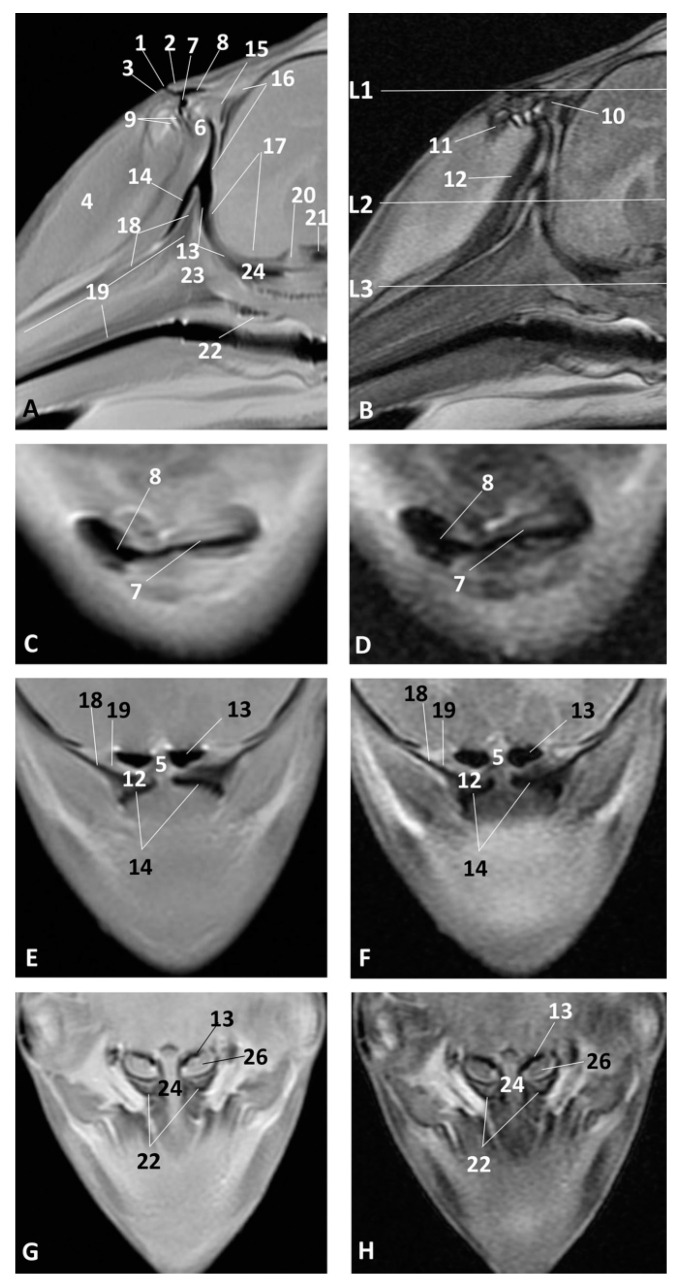
Images of the external nose and nasal cavity. MR sagittal images (**A**,**B**) are oriented so that the rostral is to the left and the dorsal to the top. MR coronal images (**C**,**H**) are oriented so that the rostral is to the bottom and caudal to the top. (**A**) T1-weighted SE sagittal plane, (**B**) T2-weighted FrFSE sagittal plane. (**C**,**E**,**G**) T1-weighted SE coronal plane. (**D**,**F**,**H**) T2-weighted FrFSE coronal plane. (**C**,**D**) Level 1. (**E**,**F**) Level 2. (**G**,**H**) Level 3. 10 months, dde14. 1, Rima naris; 2, Upper lip; 3, Lower lip; 4, Melon; 5, Nasal septum: bony part; 6, Nasal plug; 7, Nasal cavity: vestibule; 8, Vestibule: nasal diverticulum; 9, Vestibule: nasal accessory diverticulum (nasofrontal sac); 10, Caudal vestibular muscles; 11, Rostral vestibular muscles; 12, Nasal plug muscles; 13, Nasal cavity: respiratory part; 14, Nasal cavity: incisive recess; 15, Nasal bone; 16, Frontal bone; 17, Ethmoid bone: perpendicular lamina; 18, Incisive bone; 19, Maxillary bone; 20, Presphenoid bone; 21, Basisphenoid bone; 22, Pterygoid bone; 23, Vomer bone; 24, Mesorostral cartilage; 25, Choanae; 26, Pharyngeal muscles.

In the common dolphin (*Delphinus delphis*) fetus, ten months old, we could see the lumen of the vestibule and of the nasal (vestibular sacs) and accessory diverticula (nasofrontal sacs), all hypointense in T1 and T2. Also, the nasal plugs, the vestibular folds and their associated muscles are moderately hyperintense in T1 and hypointense in T2 (Figure 13A–D). The respiratory part and incisive recess are hypointense in T1 and T2. The incisive recess is surrounded rostrally by the nasal plug muscles and caudally by the incisive and maxillary bones (Figure 13A–D). This recess could also be observed in the coronal slices extending to the choanae (Figure 13E–H). The majority of the bones forming the nasal cavity have been identified (Figure 13A,B,E,H).

### 3.3. Computed Tomography and 3D Reconstruction of Bony Nasal Cavity

The 3D reconstruction of the skull bones has been performed in order to show nasal cavity development from early fetal stages where the mesenchymal tissue is abundant and the bones of the skull are being formed by intramembranous and endochondral ossification.

**Figure 14:**
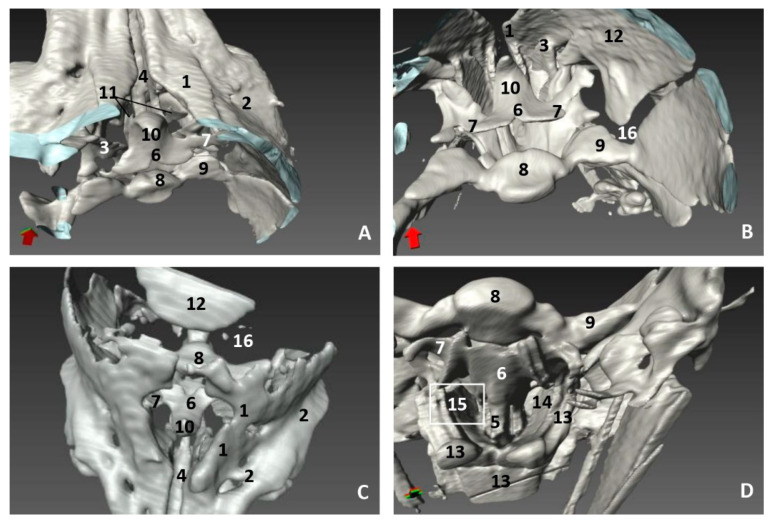
3D reconstruction images of bony nasal cavity using AMIRA and PET/SPEC/CT. 3.5 months, dde2. (**A**) *Dorsal view.* (**B**) *Caudal view.* (**C**) *Dorsal view.* (**D**) *Ventral view*. 1, Incisive bone; 2, Maxillary bone; 3, Nasal face of maxillary bone; 4, Vomer bone: groove; 5, Vomer bone: ventral crest; 6, Presphenoid bone: body; 7, Presphenoid bone: wings; 8, Basisphenoid bone: body; 9, Basisphenoid bone: wings; 10, Ethmoid bone; 11, Maxillary bone (nasal face): ossification nuclei; 12, Frontal bone: cerebral face; 13, Pterygoid bone; 14 Palatine bone; 15, Choanae; 16, Fontanelles.

In the less developed second fetus (dde2), we could see the main bones forming the rostral part of the bony nasal cavity such as the incisive and maxillary bones and the vomer groove dividing both cavities (Figure 14A,B). The presphenoid (body) and the ethmoid (body) bones are in close contact from an early developmental stage, and could only be distinguished as separate using MRI (Figure 10). In a dorsal view, we observed the bones of the braincase but not the bones separating the nasal and the cranial cavities (Figure 14C). The choanae were observed formed by the palatine and pterygoid bones and divided ventrally by the vomer crest (Figure 14D).

**Figure 15:**
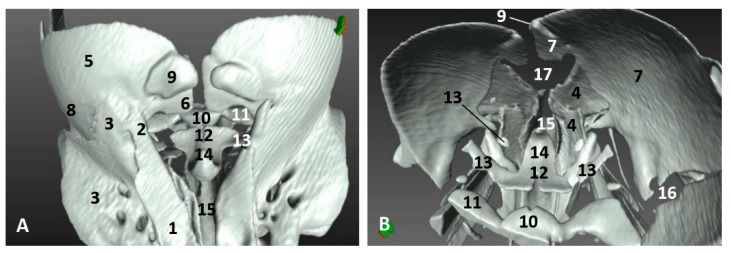
3D reconstruction images of bony nasal cavity using AMIRA and PET/SPEC/CT. 4.5 months, scop1. (**A**) *Rostral view.* (**B**) *Caudal view.* 1, Incisive bone; 2, Incisive bone: nasal process; 3, Maxillary bone: external face; 4, Maxillary bone: nasal face; 5, Frontal bone: external face; 6, Frontal bone: nasal face; 7, Frontal bone: cerebral face; 8, Temporal bone; 9, Nasal bone; 10, Basisphenoid bone: body; 11, Basisphenoid bone: wings; 12, Presphenoid bone: body; 13, Presphenoid bone: wings; 14, Ethmoid bone; 15, Vomer bone: groove; 16, Fontanelles; 17, Maxilloincisive fissure (bony naris opening).

A more developed striped dolphin (*Stenella coeruleoalba*) fetus, scop1, showed the frontal bone growing towards the incompletely-ossified ethmoid bone (Figure 15A). The rostral wall of the nasal cavity, mainly formed by the maxillary bone, is closing towards the vomer. The ethmoid bone (ethmoidal fossa) remains unossified and the bones at the base of the skull are quite separated (Figure 15B).

**Figure 16:**
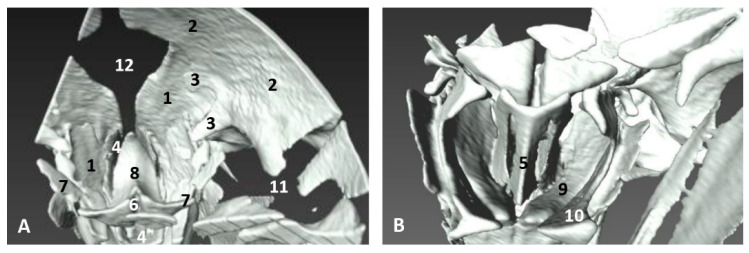
3D reconstruction images of bony nasal cavity using AMIRA and PET/SPEC/CT. 5.5 months, dde5. (**A**) *Caudal view.* (**B**) *Ventrocaudal view*. 1, Maxillary bone: nasal face; 2, Frontal bone: cerebral face; 3, Frontal bone: wall projections; 4, Vomer bone: groove; 5, Vomer: ventral crest; 6, Presphenoid bone: body; 7, Presphenoid bone: wings; 8, Ethmoid bone: crista galli; 9, Palatine bone; 10, Pterygoid bone; 11, Fontanelles; 12, Maxilloincisive fissure (bony naris opening).

At five and a half months, the fetus (dde5) shows an unossified ethmoidal fossa and bony projections from the frontal bone towards the ethmoid bone were observed (Figure 16A). The choanae are clearly seen, but there are still areas lacking ossification (Figure 16B).

**Figure 17:**
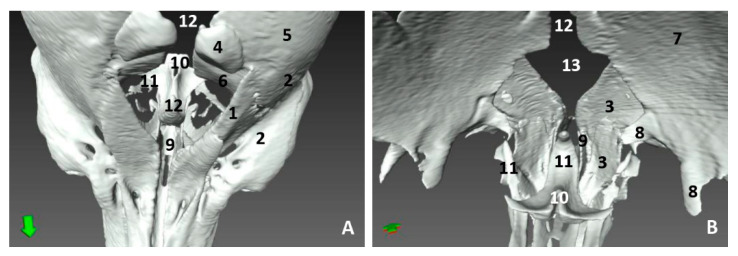
3D reconstruction images of bony nasal cavity using AMIRA and PET/SPEC/CT. 5.8 months, dde6. (**A**) *Dorsal view.* (**B**) *Caudal view.* 1, Incisive bone: external face; 2, Maxillary bone: external nasal face; 3, Maxillary bone: nasal face; 4, Nasal bone; 5, Frontal bone: external face; 6, Frontal bone: nasal faces; 7, Frontal bone: cerebral face; 8, Frontal bone: wall projections; 9, Vomer bone: groove; 10, Presphenoid bone: body; 11, Presphenoid bone: wings; 12, Ethmoid bone; 12, Fontanelles; 13, Maxilloincisive fissure (bony naris opening).

In the next common dolphin (*Delphinus delphis*) fetus of almost 6 months’ gestation, dde6, the rostral wall of the nasal cavity is almost closed. The maxilloincisive fissure is almost formed and fontanelles are decreasing in size (Figure 17A,B).

**Figure 18:**
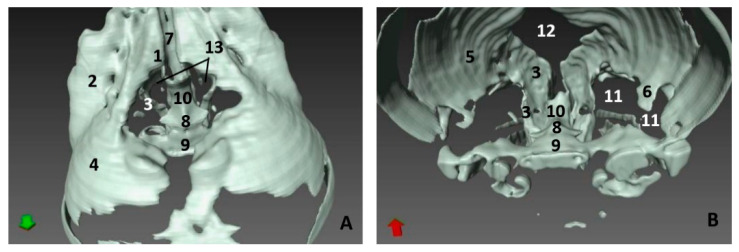
3D reconstruction images of bony nasal cavity using AMIRA and CT. 9 months, dde13. (**A**) *Dorsal view.* (**B**) *Caudal view.* 1, Incisive bone; 2, Maxillary bone: external nasal face; 3, Pterygoid bone; 4, Frontal bone: external face; 5, Frontal bone: cerebral face; 6, Frontal bone: wall projections; 7, Vomer bone: groove; 8, Basisphenoid bone; 9, Presphenoid bone; 10, Ethmoid bone: crista galli; 11, Fontanelles; 12, Maxilloincisive fissure (bony naris opening); 13, Nasal cavities.

In the common dolphin (*Delphinus delphis*) fetus of 9 months’ gestation, dde13, the walls of the nasal cavities, near to the choanae, are closing as is the rostral wall, though there are still mesenchymal areas (Figure 18A,B).

**Figure 19:**
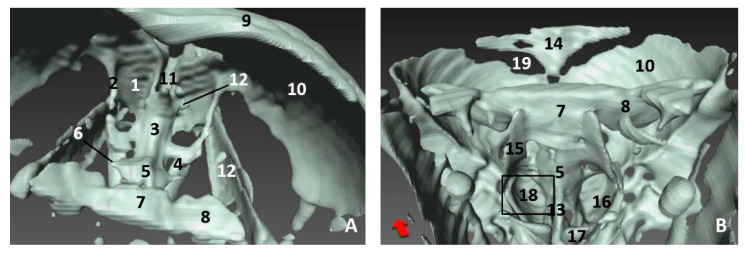
3D reconstruction images of bony nasal cavity using AMIRA and CT. 10 months, dde14. (**A**) *Caudal view.* (**B**) *Ventrocaudal view.* 1, Maxillary bone: nasal face (rostral nasal wall); 2, Maxillary bone: lateral nasal projections; 3, Ethmoid bone; 4, Ethmoid bone: ossification area (lamina cribosa); 5, Presphenoid bone: body; 6, Presphenoid bone: wings; 7, Basisphenoid bone: body; 8, Basisphenoid bone: wings; 9, Frontal bone: external face; 10, Frontal bone: cerebral face; 11, Vomer bone: groove; 12, Vomer bone: wings; 13, Vomer bone: ventral crest; 14, Interparietal bone; 15, Basisphenoid bone: pterygoid crest; 16, Palatine bone; 17, Pterygoid bone; 18, Choanae; 19, Fontanelles.

In the common dolphin (*Delphinus delphis*) fetus, 10 months of gestation, dde14, the rostral wall of the nasal cavity is totally closed by the maxillary bone, forming the bony walls in both cavities at both sides of the ethmoid bone. The lamina cribosa and the vomer wings are incomplete. The choanae are also seen well delimited (Figure 19A,B).

**Figure 20:**
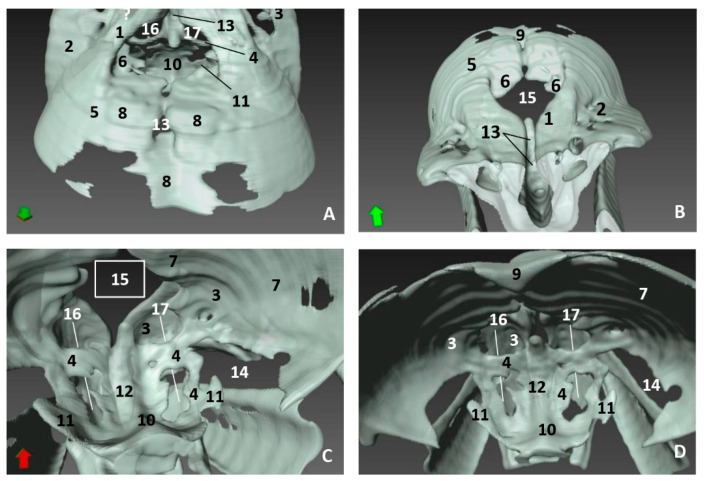
3D reconstruction images of bony nasal cavity using AMIRA and CT. newborn, scomu1. (**A**) *Dorsal view.* (**B**) *Frontal view.* (**C**,**D**) *Caudal view.* 1, Incisive bone; 2, Maxillary bone: external face; 3, Maxillary bone: nasal face; 4, Ethmoid bone: ossification nuclei (lamina cribosa); 5, Frontal bone: external face; 6, Frontal bone: nasal face; 7, Frontal bone: cerebral face; 8, Nasal bone; 9, Interparietal bone; 10, Presphenoid bone: body; 11, Presphenoid bone: wings; 12, Ethmoid bone: crista galli; 13, Ethmoid bone: lamina perpendicular; 14, Fontanelles; 15, Maxilloincisive fissure (bony naris opening); 16, Left nasal cavity; 16, Right nasal cavity.

The newborn striped dolphin (*Stenella coeruleoalba*), scomu1, shows the caudal wall of the nasal cavity almost closed, with bony projections from the frontal and maxillary bones towards the ethmoid bone. (Figure 20A,B). The lamina cribosa of the ethmoid bone is closing the wall of right and left nasal cavities (Figure 20C,D).

**Figure 21:**
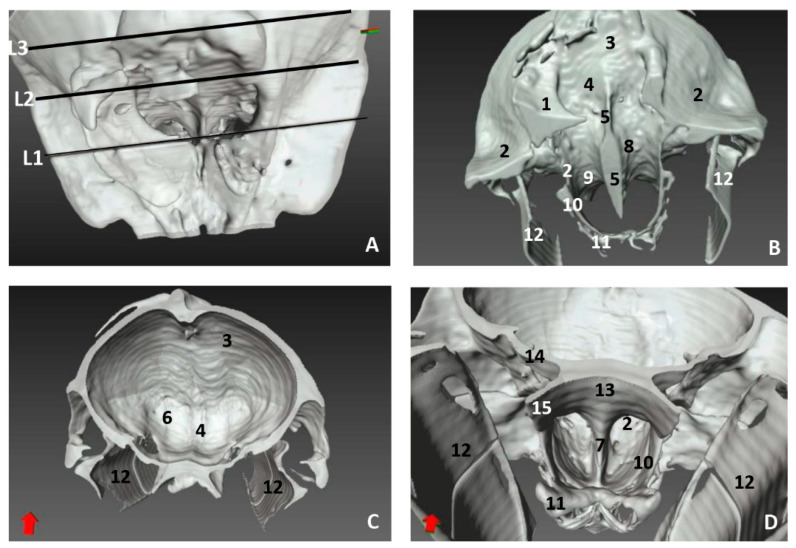
3D reconstruction images of bony nasal cavity using AMIRA and CT. newborn, scomu1. (**A**) Level sections. *Dorsal view*; (**B**) Level 1 (L1), *Rostral view*; (**C**) Level 2 (L2), *Caudal view*; (**D**) Level 3 (L3), *Ventrocaudal view*. 1, Incisive bone; 2, Maxillary bone; 3, Frontal bone; 4, Ethmoid bone; 5, Ethmoid bone: perpendicular lamina; 6, Ethmoidal fossa: lamina cribosa; 7, Vomer bone: ventral crest; 8, Vomer bone: wings; 9, Presphenoid bone: wings; 10, Palatine bone; 11, Pterygoid bone; 12, Mandibles; 13, Basisphenoid bone: body; 14, Basisphenoid bone: wings; 15, Basisphenoid bone: pterygoid crest.

In the adult striped dolphin (*Stenella coeruleoalba*), three sections of the nasal cavity have been performed (Figure 21A). We observed the bony nasal septum formed by vomer and perpendicular lamina of ethmoid bone and the closed caudal wall of the nasal cavity (Figure 21B) formed by the ethmoidal and cerebral fossae (Figure 21C). In the last section we could see the completely defined caudal region of the nasal cavities (Figure 21D). Several vestigial perpendicular fissures were observed in the ethmoidal fossae and a small cribriform area in the nasal aspect of the ethmoid bone.

### 3.4. Computed Tomography and 3D Reconstruction of Nasal Cavity Spaces

**Figure 22:**
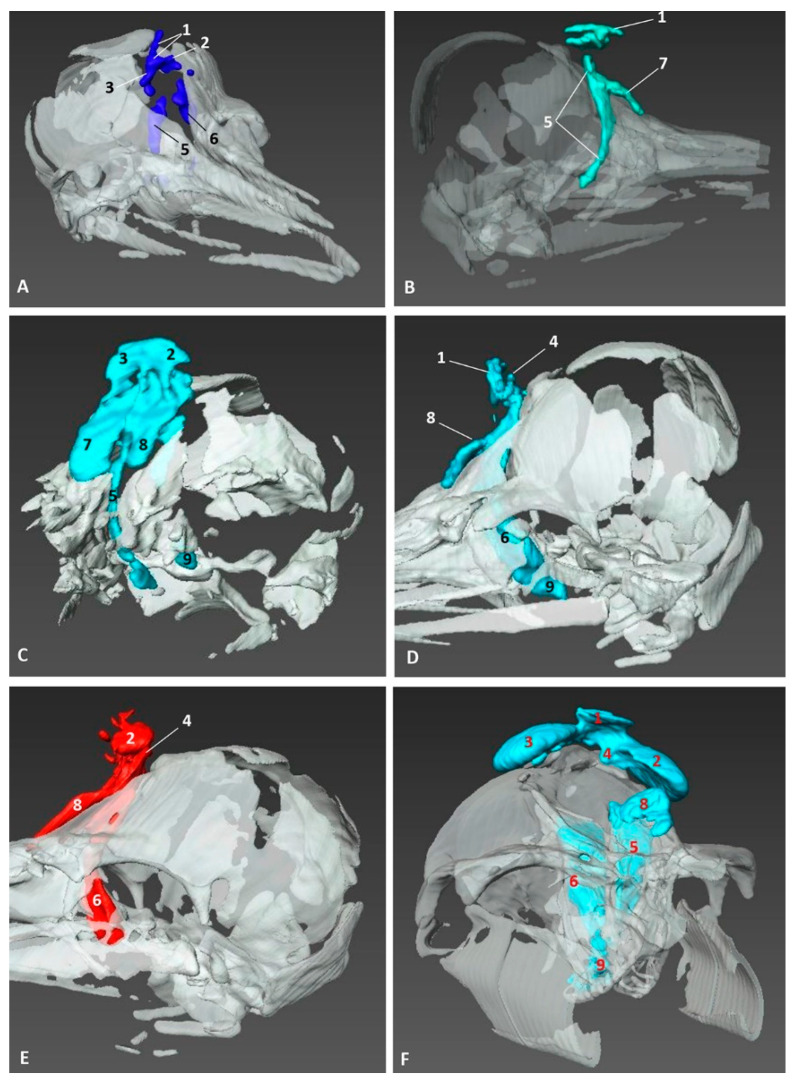
Amira 3D reconstructions of nasal cavity spaces after injecting silicone. Hiperattenuated CT images and air spaces were used to obtain the internal endocast. (**A**) *Right rostral aspect*. 3.5 months, dde2. (**B**) *Right lateral aspect*. 4.5 months, scop1. (**C**) *Left rostral aspect*. 5 months, gma1. (**D**) *Left lateral aspect*. 5.8 months, dde6. (**E**) *Left lateral aspect*. 10 months, dde14. (**F**) *Left rostral aspect*. Adult, scomu5. 1, Nasal cavity: vestibule; 2, Vestibule: left diverticulum; 3, Vestibule: right diverticulum; 4, Vestibule: left accessory diverticulum; 5, Nasal cavity: right respiratory part; 6, Nasal cavity: left respiratory part; 7, Nasal cavity: right incisive recess; 8, Nasal cavity: left incisive recess; 9, Choanae.

In order to give a better perspective of the respiratory cavity, 3D casts were observed in both lateral and oblique views along with the 3D reconstructions of the skull bones. In the second less developed common dolphin (*Delphinus delphis*) fetus, dde2, we injected a small amount of silicone, with similar properties to CT iodinate contrast medium, to enable us to obtain an endocast and dilate the cavities to make the 3D reconstruction. Within the vestibule we could already distinguish the bilateral nasal diverticula. The accessory diverticulum was not clearly seen. We could observe the endocast of respiratory and olfactory part of the nasal cavity (Figure 22A). A more developed striped dolphin (*Stenella coeruleoalba*) fetus, scop1, showed the incisive recesses extending to the snout base overlapping the incisive bone (Figure 22B). In the pilot whale (*Globocephala melas*) fetus of five months’ gestation, gma1, we could observe the expansion of the incisive recesses as the fetus is developing (Figure 22C). In common dolphin (*Delphinus delphis*) fetus of 5.5 months’ gestation, dde5, the left lateral view allowed us to distinguish the accessory nasal diverticulum, which was very small and oriented rostrally (Figure 22D). In the last studied fetus, dde14, we observed the accessory nasal diverticulum ventral to the left nasal diverticulum (Figure 22E). In the adult striped dolphin (*Stenella coeruleoalba*) specimen, scomu1, we saw well-dilated left and right diverticula, the left accessory diverticulum, left incisive recess and parts of the left respiratory and olfactory parts of the nasal cavity. The right accessory diverticulum and right incisive recess were not dilated by air or the injected silicone, so it could not be reconstructed (Figure 22F).

### 3.5. Study of the Nasal Cavity Using Sectional Anatomy and Dissection Techniques

#### 3.5.1. Sectional Anatomy

The sagittal sections were performed in both a juvenile and an adult striped dolphin (*Stenella coeruleoalba*). The coronal sections were performed in a newborn striped dolphin (*Stenella coeruleoalba*) covering the whole nasal cavity. The two first sections are placed in the vestibule of the nasal cavity while the other four are in the respiratory and olfactory part of the nasal cavity.

**Figure 23:**
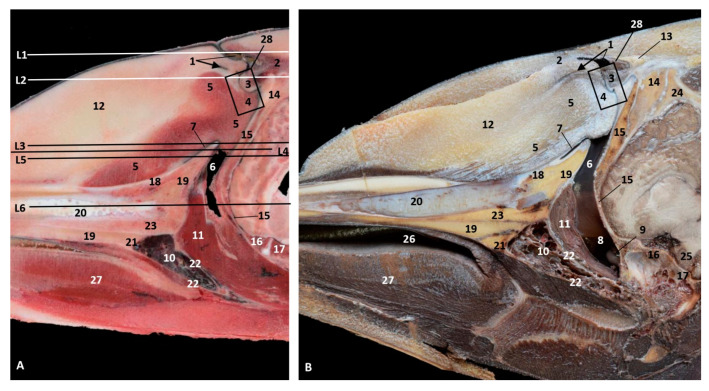
(**A**,**B**) Sagittal sections of nasal cavity. Sagittal sections images are oriented so that the rostral is to the left and the caudal to the right. (**A**) Level sections (L1–L6), Fresh section, juvenile, scomu3. (**B**) Fixed section, adult, scomu6. 1, Nasal cavity: vestibule (line) and (arrow) accessory nasal diverticulum; 2, Upper lip and vestibular fold muscles; 3, Vestibular fold; 4, Nasal plug; 5, Nasal plug and lower lip muscles; 6, Nasal cavity: respiratory part; 7, Nasal cavity: incisive recess; 8, Choanae; 9, Aditus laryngis; 10, Pterygopalatine recess: pharyngeal recess of pterygoid and palatine bones; 11, Palatopharyngeal muscles (sectioned); 12, Melon; 13, Nasal bone; 14, Frontal bone; 15, Ethmoid bone; 16, Presphenoid bone; 17, Basisphenoid bone; 18, Incisive bone; 19, Maxillary bone; 20, Mesorostral cartilage; 21, Palatine bone; 22, Pterygoid bone; 23, Vomer bone; 24, Interparietal bone; 25, Hypophysis; 26, Oral cavity; 27, Tongue; 28, “Monkey or phonic lips”.

At the level of the vestibule we could see many vestibular folds, together with the nasal plugs forming the “monkey lips” involved in sound production. We also observed muscles of the external nose, which enable the opening of its caudal lip. We also could see the accessory diverticulum and the vestibule (Figure 23A,B).

**Figure 24:**
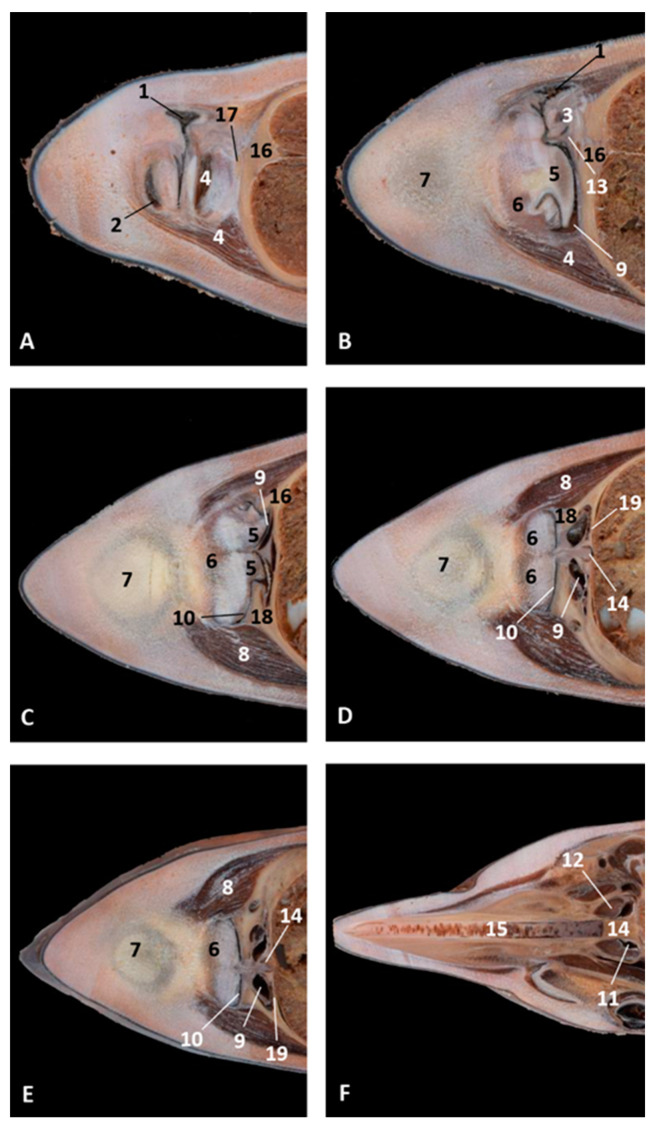
Coronal sections of nasal cavity. Coronal sections images are oriented so that the rostral is to the left and the caudal to the right. (**A**,**B**) Level 1 and 2. Nasal cavity: vestibule. (**C**–**F**) Levels 3 to 6. Nasal cavity: respiratory and olfactory parts. Newborn, scomu2. 1, Vestibule: right diverticulum; 2, Vestibule: left diverticulum; 3, Vestibular folds; 4, External nose muscles; 5, Nasal plugs; 6, Nasal plug muscles and connective tissue; 7, Melon; 8, Melon muscles; 9, Nasal cavity: respiratory part (nasal mucosae); 10, Nasal cavity: incisive recess; 11, Choanae; 12, Pharyngeal muscles; 13, Nasal septum: membranous part; 14, Nasal septum: bony part (vomer); 15, Mesorostral cartilage; 16, Frontal bone; 17, Nasal bone; 18, Incisive and maxillary bones; 19, Ethmoid bone.

The coronal sections at the vestibule level showed the left and right diverticulum and nasal plugs close to the vestibular fold (Figure 24A,B).

#### 3.5.2. Dissections

##### Dolphin Specimens

The dissections were performed in a newborn (scoce1) stranded in the Mediterranean coast of Ceuta and two adults (scomu5 and scomu7) stranded in the Mediterranean coast of Murcia.

**Figure 25:**
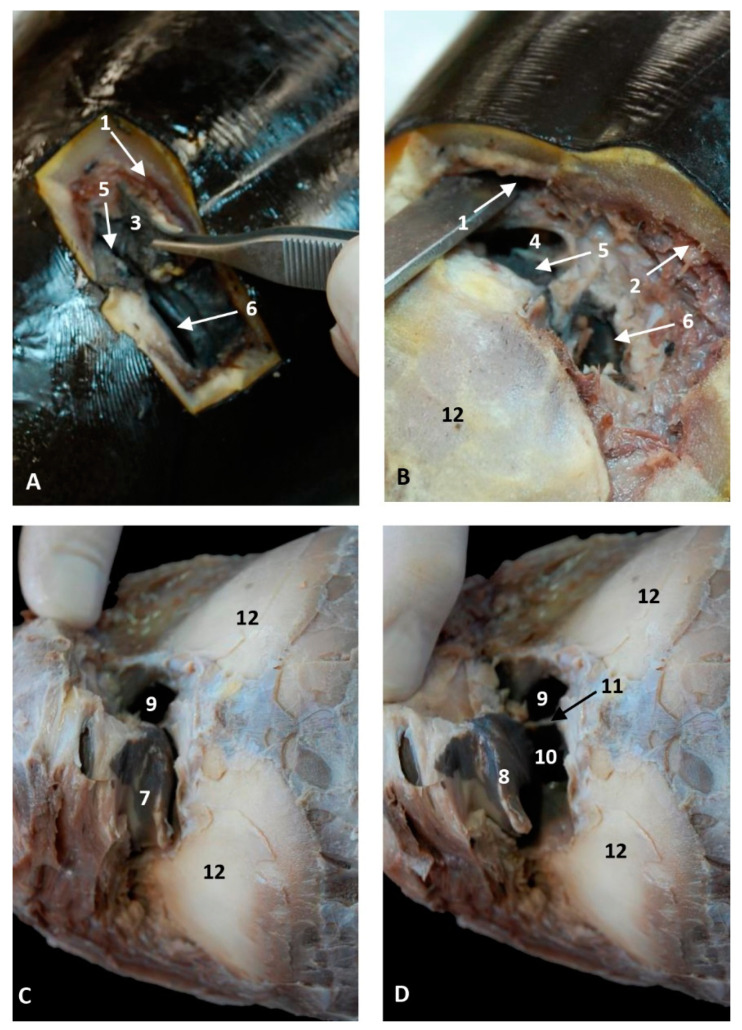
Dissection of nasal cavity. (**A**,**B**) Head is oriented so that the rostral side of the head is to the right up corner and caudal is to the left down corner. (**C**,**D**) Head is oriented so that the rostral to the left and the caudal to the right. scoce1. (**A**) Right nasal cavity: vestibule. Clogged right nasal cavity (hyperkeratosis). (**B**) Nasal cavity: vestibule after removed melon skin. (**C**,**D**) Nasal plug opened and closed. Newborn, scoce1. 1, Nasal cavity: right diverticulum; 2, Nasal cavity: left diverticulum; 3, Nasal cavity: accessory diverticulum opened; 4, Accessory diverticulum opened and mucosa partly removed; 5, Right nasal plug clogged; 6, Left nasal plug normal but hypertrophied; 7, Left nasal plug closed; 8, Left nasal plug opened; 9, Right nasal cavity: respiratory part; 10, Left nasal cavity: respiratory part; 11, Nasal septum: bony part (lamina perpendicular ethmoid bone); 12, Frontal bones.

After removing the external nose in the newborn, we observed dorsally the right nasal diverticulum and ventrally the accessory nasal diverticulum caudal to the right nasal plug (Figure 25A). The right nasal plug was clogged and the left one was normal (Figure 25B). We also observed two functional positions of the left nasal plug, one opened and two closed, and the nasal cavities divided by the nasal septum (Figure 25C,D).

**Figure 26:**
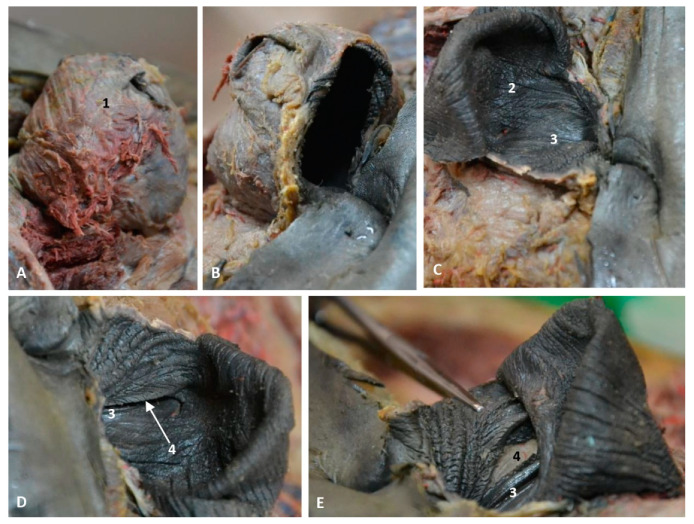
Dissection of nasal cavity: vestibule. Detailed images of a nasal diverticulum. Adult, scomu5. (**A**) Nasal cavity: vestibule. Right diverticulum dilated. *Right lateral aspect*. (**B**) Nasal cavity: vestibule. Right diverticulum partially sectioned. *Rostral view*. (**C**) Right diverticulum opened. *Rostral view*. (**D**) Right diverticulum opened. *Caudal view*. (**E**) Right diverticulum border displaced dorsally. *Caudal view*. 1, Nasal cavity: right diverticulum (dilated); 2, Right diverticulum: mucosa; 3, Nasal plug; 4, Accessory diverticulum.

In a preserved female striped dolphin (*Stenella coeruleoalba*), scomu5, the right nasal diverticulum was dilated to better understand its size and form (Figure 26A). In subsequent images, the rostral wall of diverticulum has been removed to show the dark mucosa and ventrally the closed nasal plug (Figure 26B,C). The final images show in detail the closed nasal plug and the access to the accessory diverticulum, first closed (Figure 26D) and then opened showing a portion of the less pigmented mucosa (Figure 26E).

**Figure 27:**
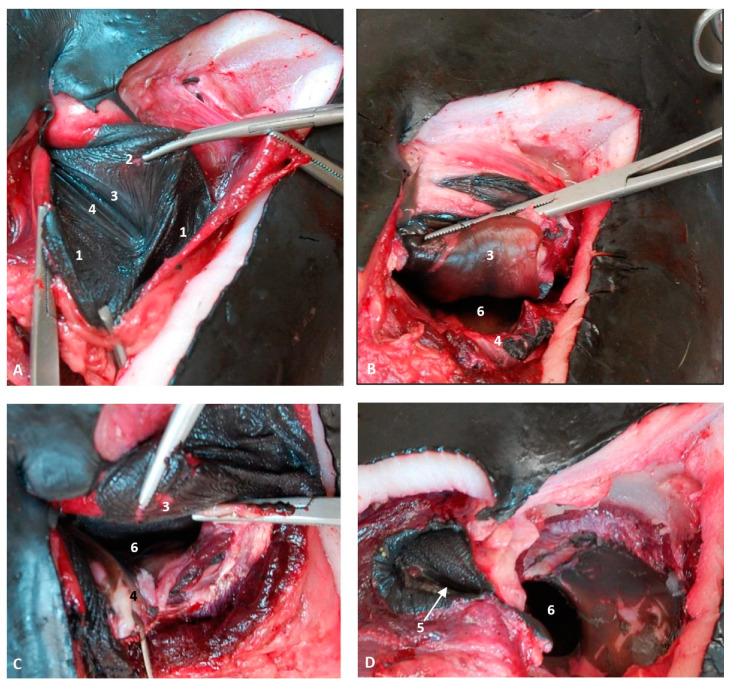
Dissection of nasal cavity: vestibule and respiratory part. (**A**,**D**) Head is oriented so that the rostral side of the head is to the top and caudal is at the bottom. Adult, scomu7. (**A**) Right nasal cavity: vestibule open. (**B**) Right nasal cavity: nasal plug displaced rostrally. (**C**) Right nasal cavity: nasal plug displaced rostrally and vestibular fold caudally. (**D**) Right nasal plug has been removed and left is observed closed. 1, Nasal cavity: right diverticulum; 2, Accessory diverticulum opened; 3, Right nasal plug; 4, Vestibular fold; 5, Left nasal cavity: left nasal plug; 6, Right nasal cavity: respiratory part.

In a female fresh adult striped dolphin (*Stenella coeruleoalba*), scomu7, we could identify the right and the accessory diverticula. The mucosa of both diverticula is dark in colour. The right nasal plug is tightly closed (Figure 27A). The nasal plug has been displaced rostrally showing the respiratory part of the nasal cavity (Figure 27B). The vestibular fold and nasal plug have been displaced caudally and rostrally, respectively, and inside the respiratory part of the nasal cavity we could observe the dark nasal mucosa (Figure 27C,D). Finally, we examined in detail the closed entrance to the left nasal plug and the respiratory part of the right nasal cavity which was patent after removal of the nasal plug (Figure 27D).

###### Horse Specimens

The dissections were performed in one foal fetus (ecal1) and two adult (ecal2 and 3) horses from the slaughter house (Alicante).

**Figure 28:**
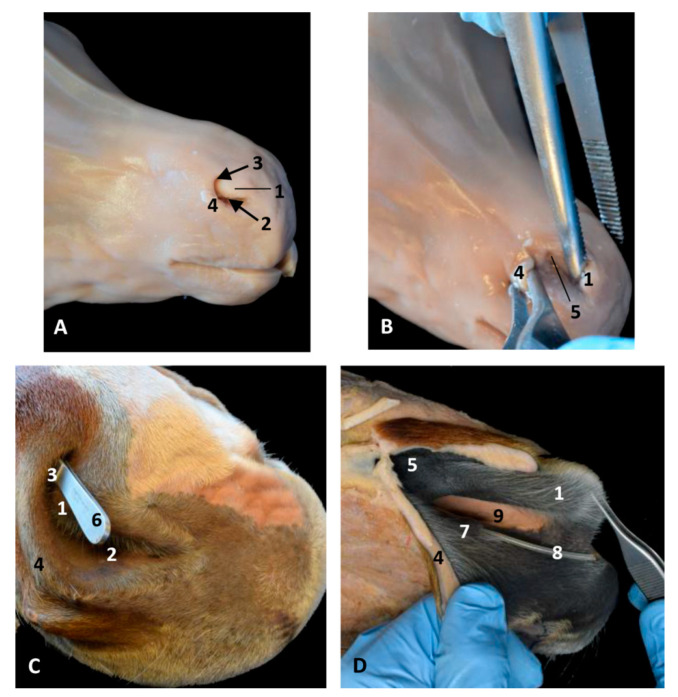
Nose dissections at right nasal vestibule level in a foal fetus (**A**,**B**) and two adult horses. (**C**,**D**). (**B**) Wing of the nostril has been retracted laterally to observe the nasal diverticulum. (**C**) Forceps are inside the nasal diverticulum. (**D**) The nose has been dissected to observe pigmentation differences between mucosa of the vestibule and the respiratory part of the nasal cavity. (**A**–**D**) *Right lateral view*. 1, Alar fold; 2, Nostril; 3, False nostril; 4, Wing of the nostril; 5, Nasal diverticulum; 6, Forceps; 7, Nasal vestibule: mucosa; 8, Nasolacrimal orifice (plastic tube); 9, Nasal cavity: respiratory part.

The fetal vestibule was observed in its anatomical position showing the alar fold and nostril (false nostril is the portion of the nostril continuous with the nasal diverticulum) which is the orifice of nasal vestibule (Figure 28A–C). Dissections showed the differences between the mucosa of nasal diverticulum, vestibule, alar fold and respiratory part of the nasal cavity (Figure 28B–D).

### 3.6. Histological Study of the Nasal Cavity

#### 3.6.1. Dolphin

**Figure 29:**
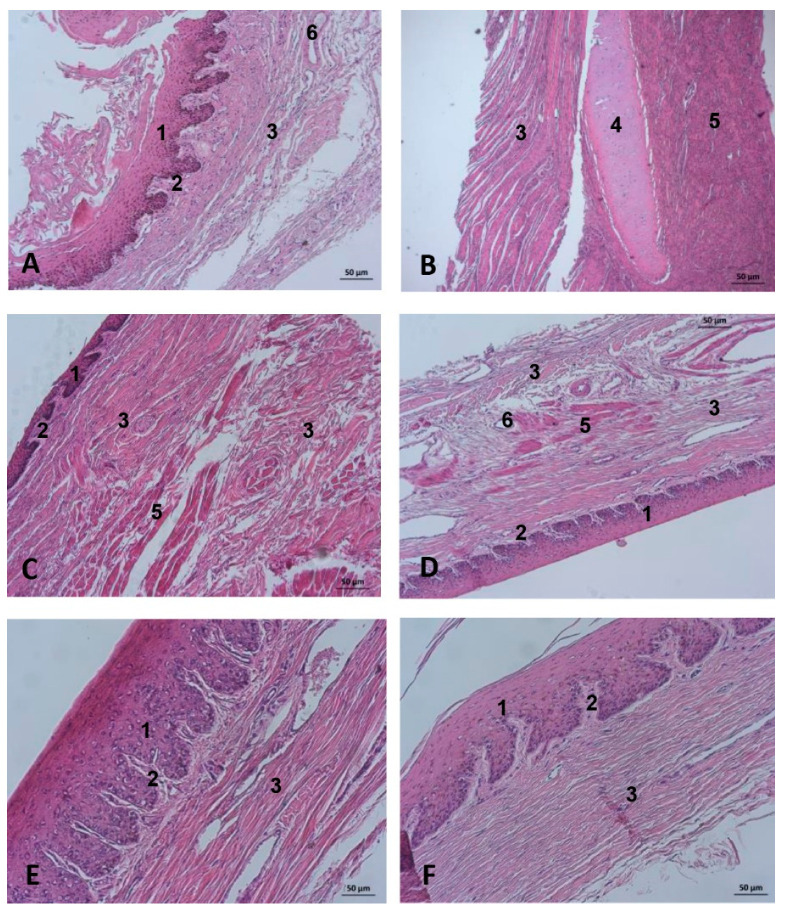
Histological study of nasal mucosa: vestibule, respiratory and olfactory parts. H-E staining technique. Adult, scomu6. (**A**) Left diverticulum, 10×. (**B**) Vestibular fold, 10×. (**C**) Nasal plug, 10×. (**D**) Respiratory part, 10×. (**E**) Olfactory part, 20×. (**F**) Incisive recess, 20×. 1, Epithelium: stratified squamous keratinized and pigmented; 2, Papillary layer; 3, Connective tissue base; 4, Cartilage; 5, Striated muscular base; 6, Vessels.

The vestibular mucosa, specifically its diverticulae, is a stratified squamous epithelium, both pigmented and keratinized. The papillary layer is wide. The connective tissue base is normal and contains small vessels (Figure 29A). The vestibular folds show a connective tissue base with cartilage and striated muscle (Figure 29B). The nasal plug has a stratified epithelium, narrow and pigmented with a papillary layer wide and flattened. It has stratified musculature in discontinued fascicles. Its connective tissue base is strong with muscular fascicles (Figure 29C). The mucosa in the respiratory part has a pseudostratified arrangement but cilia were not observed (10 layers). A deep and narrow papillary layer is observed. A dense connective tissue base with a few muscular fibers and blood vessels was seen (white areas enlarged and empty) (Figure 29D). The mucosa of the olfactory part is a pseudostratified epithelium but cilia were not seen and it was more rounded and wider than the respiratory part (15–16 layers). The papillary layer is narrow and vascularized with a connective tissue base (Figure 29E). A stratified squamous epithelium lines the incisive recess. The papillary layer is wide with little vascularization. The connective tissue base containing some nerves was seen (Figure 29F).

#### 3.6.2. Horse

**Figure 30:**
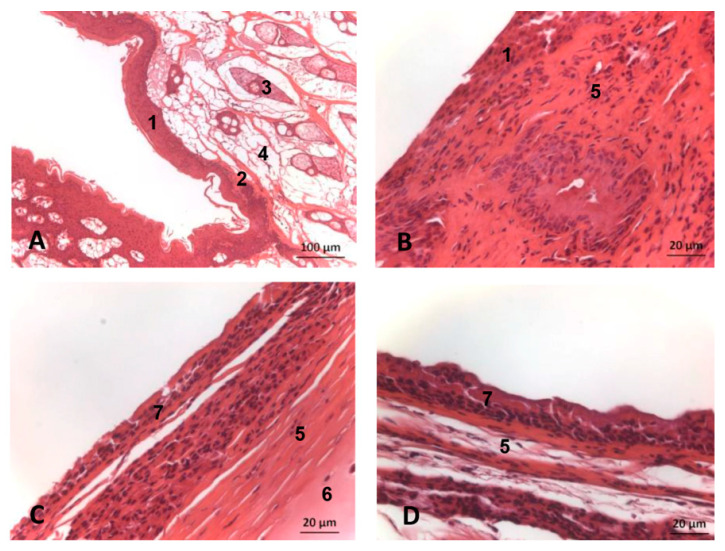
Histological study of nasal mucosa: vestibule, respiratory and olfactory parts. H_E stain technique. Adult Ecal4 (**A**) Nasal vestibule 10×. (**B**) Alar fold 40×. (**C**) Respiratory part. 40×. (**D**) Olfactory part. 20×. 1, Epithelium: stratatified squamous keratinized and pigmented; 2, Papillary stratum; 3, hair; 4, Fat tissue; 5, Connective tissue base; 6, Cartilage; 7, Respiratory epithelium.

The vestibular mucosa of the nasal cavity, specifically its diverticulae, is a stratified squamous epithelium, both pigmented and keratinized with hair and with associated sebaceous glands. The papillary layer is not well defined. The connective tissue base contains some adipose tissue (Figure 30A). The alar fold has a stratified squamous epithelium which is not keratinized. The connective tissue base is large and dense. There are groups of glands between the sebaceous tissue (Figure 30B). The respiratory part of the nasal cavity has a pseudostratified epithelium, forming a cylindrical mucosa with cilia and a cartilaginous base (Figure 30C). The mucosa of the olfactory part is a pseudostratified epithelium but olfactory cilia were not clearly seen (Figure 30D).

## 4. Discussion

### 4.1. Anatomical, Comparative and Functional Commentaries

#### 4.1.1. External Nose

The external nose of adult odontocetes and mysticetes is well described [10,11,15,16,19,21,25,26,41,42,43,44] but the information is sometimes confusing, often due to differing terminology. Our study goes from the first development stages of the fetus to the adult stage.

The endoscopy technique to analyze the nasal cavity has allowed us to observe the external nose and nasal cavity morphology caudal to the choanae. In mammals, the external nose is part of the face rostral to the frontal region and dorsal to the infraorbital, buccal and oral region [38]. We have observed the highly characteristic morphology of the cetacean nose with the endoscopy images. The presence of nasal lips (rostral and caudal) and its anatomical position, closed from the beginning of development to avoid the entrance of water and salt into the nasal cavity is one of the first image proofs of the development of the two prominences in the caudal lip and their division by a median groove from the nasal septum to the caudal lip. During early stages of fetal development the external nose and the melon were observed as a common anatomical area delimited by a line and covered by an epidermis paler than the rest of the head. Together with the function of the melon in the projection of sounds produced by “phonic lips” [6,43,44], this supports our idea that the melon is forming part of the external nose both anatomically and functionally, and not merely as a part of the nasal complex. 

These changes in morphology will be helpful in determining the stages of fetal development. 

The MRI and dissection images allowed us to observe, as well as other authors [19,25,26,41] that both the caudal and the rostral lips present retractor muscles which act to open the nose for breathing when the animal rises above the water. Also, we have observed that the most retracted lip was the caudal one. 

We also discovered that closing of the lips becomes more airtight as development progresses to adulthood, whose particular anatomy forces these lips to double their size with an increased number of striations, rendering them more waterproof.

#### 4.1.2. Nasal Cavities

The nasal cavity of odontocetes is similar to that of mysticetes except in the sperm whale, a species with a unique morphology [13,18,42].

The odontocete nasal cavity has only one nostril (naris) and one vestibule with paired diverticula, and two respiratory and olfactory parts divided by a nasal septum and ending caudally at the choanae. The equine nose has two nostril, two vestibules of the nose and a cutaneous blind sac (diverticulum nasi) [38]. Except for the sperm whale, the cetacean nasal cavity has a vertical orientation and the external nose and melon are located dorsally [6] with the choanae ventrally, and as described before, the nasal bones are retracted towards the frontal bones [1,2,3,15]. Therefore, we suggest that the cetacean nasal cavity should be more properly called the maxillary cavity.

##### Vestibule

The nasal cavity vestibule is a space described in all the terrestrial mammals as the hall of the nasal cavity rostral to therespiratory and olfactory part. Though theoretically it is a simple part of this cavity, most of the studies define it in the bottlenose dolphin as a very complex area [9,15] and in our opinion it is not fully explained in previous publications. The presence of two diverticula of the nasal vestibule is clearly shown by endoscopy, 3D reconstructions, sections and dissection studies. These diverticula present a pigmented mucosa as in other species [18] and also in the dissections, sections and histology of our study. The stratified squamous epithelium is similar to that of the equine nose and corroborates our idea about its function, which is protection from external agents and water [17,28]. The vestibular fold shows a very different mucosa from that of the respiratory region, probably related to is proposed function in sound production. Endoscopy permitted us to observe the morphology of the right and left diverticula as spaces with many folds and an anfractuous arrangement as in other cetacean species [17]. The nasal endocast and the volumetric reconstructions enabled us to study its morphology and to locate another accessory nasal diverticulum that we confirmed by dissections. Though we know the bottlenose dolphin vestibule morphology as very complex [9,15,26], our developmental study shows that the vestibule in common and striped dolphins *Stenella coeruleoalba* and pilot whale *Globocephala melas* is less complex.

Another important aspect of our study is the endoscopic examination of the “monkey or phonic lips” which are responsible for sound production [6,43,44]. According to our study, the dorsal parts of the sound production mechanism were the vestibular folds placed under the upper lip and the ventral parts were the nasal plugs divided by the membranous part of the nasal septum. According to our anatomical idea, the “monkey or phonic lips” are solely in charge of producing sound projected towards the melon. Diverticula, wrongly named as air sacs [7,8,9,10] because they are only present in birds in order to decrease the total weight during flight. The bronchi extend outside the lung in a form of thin-walled transparent chambers called air sacs [45] extending through the celomic cavity and bones. Diverticula avoid the entrance of possible water filtrations through the blowhole that could have entered during sound production, diving or under a stressful situation such as vessel crash or an underwater shark attack, since they are very far from the phonic lips. It is interesting to observe that vestibular folds lack an epithelium and present a connective tissue and muscular base explaining its function as sound generator.

##### Respiratory and Olfactory Parts of the Nasal Cavity

This region delivering air towards the lungs is characterized by the absence of nasal conchae or cornettes and paranasal sinuses except for the maxillary sinus [28] which is absent in the adult. The maxiloturbinate, well developed in terrestrial mammals, is vestigial in odontocetes, though there are differences between mysticetes and odontocetes [31]. If the function of heating air at this level of the nasal cavity is the main goal in the domestic mammals due to the presence of cornettes and sinuses. In cetaceans it is not the case. Also, it seems that these structures lack the olfactory function in odontocetes, as our histological analysis did not find olfactory epithelium.

##### Bony Nasal Cavity

The tomographic study of the fetal nasal cavity permitted the analysis of the interior of the cavities Volumetric reconstruction of the nasal cavity allowed us to determine the stages of ossification of the nasal cavity The maxillary bones forming the rostral wall are first to form followed by the frontal bones closing slowly towards the cranium base, but they only slowly ossify and connect with the lamina cribosa of the ethmoid bone. The final bone to ossify is the ethmoid bone, which indicates that it is to allow the passage of the olfactory nerves and form the lamina cribosa, even though the olfactory bulb is vestigial in cetaceans and persists as a remnant of phylogeny [21,30]. The vomer bone is growing rostrally slowly and we can see the groove on which the mesorostral cartilage will be placed. The bones closing the ventral floor of nasal cavity are the pterygoid and the palatine [31]. The ethmoid bones take longer to ossify and close the caudal wall of the nasal cavity which was visible in the volumetric reconstructions. The mold of the respiratory and olfactory parts showed two cavities bending caudally towards the nasopharynx surrounded by the aforementioned bones.

##### Nasal Mucosa

The endoscopic study of the nasal cavity shows at the early developmental stage a smooth mucosa which forms longitudinal folds as development progresses. We have not seen this described by other authors using other techniques, including endoscopy [34,35]. The endoscope allowed us to examine the incisive recess (premaxillary sac according to other authors [8,12,14]) that our study suggests serves to store, along with the vestibular sac [19], water escaping from the nasal vestibule which is expelled when the animal emerges to breathe [28]. The extent of the recesses could be seen in the nasal casts, as was their growth during fetal development, findings supported by the MRI data, anatomical sections and even dissection. As seen in other endoscopy studies [46] small mucosal fossae were detected close to the choanae whose function is uncertain. The nasal folds are very abundant in the adult and the lumen was narrow. Histological analysis of the nasal vestibule produced similar findings to the nasal diverticulum of the horse [7] (Figure 29A and Figure 30A) and the respiratory part had a respiratory mucosa not so similar to that of the horse), but we have found few histological studies at these levels [34,42,46]. The spherical nuclei of the sensorial cells were not observed in the olfactory part confirming the absence of olfactory function (Figure 29E).

##### Pathological Findings

The endoscopic study detected an obstruction in the right nasal cavity vestibule in the newborn from Ceuta stranded with its mother and dying afterwards. Post mortem pathologic analysis confirmed this diagnosis. Many stranded calves with respiratory problems are not fully examined due to the expense of the procedure and insufficient equipment. Using endoscopy, parasites were detected, belonging mainly to two families (*Pseudaliidae* and *Prassicaudidae*) and four genera (*Halocercus*, *Pharurus*, *Pseudalius* and *Stenurus*) [47,48]. This often undiagnosed infestation, along with the obstructions could lead to stranding of neonatal cetaceans. Except for rare cases where it is used during necropsies [46], endoscopy is restricted to live animals to examine of the lower respiratory tract (bronchis) and to diagnose the respiratory pathologies of parasitic, bacterial or fungal kind, either by direct visualization, bronchial flushes, or taking biopsies [36,37]. When necropsies are performed, we think that endoscopy of the nasal cavity should be included in necropsy protocols, despite the difficulty of the technique. 

## 5. Conclusions

Fetal anatomical endoscopic study allowed us to observe the simultaneous development of the melon and the external nose. Also, we have also seen the form and function of the external nose showing a closing formed by two lips very simple during fetal development and very sophisticated at adult stage. The vestibule showed us the “monkey lips”, two diverticula and two incisive recesses. Longitudinal mucosal folds were seen in the respiratory and olfactory parts. 3D reconstructions of nasal spaces and nasal skull gave us a spatial representation of nasal cavity development and confirmed our endoscopic observations. MRI data of sagittal and coronal sections using T1 and T2 MRI sequences were important in confirming that bony anatomical structures were correctly identified such as the presphenoid and ethmoid bone. Also, sectional anatomy and dissections aided our identification of structures seen in endoscopic, CT and MRI studies.

The histological analysis has confirmed the similarity of the dolphin nasal mucosa compared with horse nasal mucosa but cilia were not observed in the respiratory part of the nasal mucosa in odontocetes. Pathological findings which showed hyperkeratosis within the nasal vestibule should be taken into account during necropsies in stranded specimens.

The retraction of the nasal bones, the vertical position of the nasal cavities and the special bony walls leads us to recommend that “nasal cavity” should be referred to as “maxillary cavity” as these bones close the respiratory space in dolphins as nasal bones do in domestic mammals.

The nasal vestibule contains different cavities such as the nasal diverticulum which is described in horses. The cetacean diverticulum functions as a water reservoir and protection against external agents. Vestibular folds are comparable to the modified alar folds described in the horse.

The fusion between the ethmoid and presphenoid bones was seen in the second early fetal specimen. 

Finally, we conclude that the lamina cribosa of the ethmoid bone is the last bone to ossify in the nasal cavity in a newborn specimen. It shows a small perpendicular fissure at both sides of the crista galli and a small cribriform area in the nasal aspect of the ethmoid bone. Olfactory cells were not detected during histological analysis of the nasal mucosa. 

## Figures and Tables

**Table 1:** Specimens of dolphin used in this study.

Study Code	Species, Sex [29,39,40]	Anatomical, Surgical/Imaging Diagnostic and 3D Reconstruction Techniques
dde1	*Delphinus delphis* L., male fetus	Endoscopy, MRI
dde2	*Delphinus delphis* L., male fetus	Endoscopy, MRI, PET/SPEC/CT, AMIRA^®^, silicone injection
dde3	*Delphinus delphis* L., female fetus	Endoscopy, MRI
scop1	*Stenella coeruleoalba* M., female fetus	Endoscopy, MRI, PET/SPECT/CT, AMIRA^®^, silicone injection
gma1	*Globicephala melas* T., male fetus	Endoscopy, MRI, PET/SPEC/CT
dde4	*Delphinus delphis* L., male fetus	Endoscopy
dde5	*Delphinus delphis* L., female fetus	Endoscopy, MRI, PET/SPEC/CT, AMIRA^®^
dde6	*Delphinus delphis* L., female fetus	Endoscopy, PET/SPEC/CT, AMIRA^®^, silicone injection
dde7	*Delphinus delphis* L., male fetus	Endoscopy, MRI
dde8	*Delphinus delphis* L., female fetus	Endoscopy
dde9	*Delphinus delphis* L., male fetus	Endoscopy, MRI, CT, AMIRA^®^, silicone injection
dde10	*Delphinus delphis* L., female fetus	Endoscopy, MRI
dde11	*Delphinus delphis* L., male fetus	Endoscopy, MRI
dde12	*Delphinus delphis* L., male fetus	Endoscopy, MRI
dde13	*Delphinus delphis* L., female fetus	Endoscopy, MRI, CT, AMIRA^®^, silicone injection
dde14	*Delphinus delphis* L., female fetus	Endoscopy, MRI, CT, AMIRA^®^, silicone injection
scoce1	*Stenella coeruleoalba* M., male newborn	Endoscopy, Head dissection, Anatomopathological study
scomu1	*Stenella coeruleoalba* M., female newborn	CT, AMIRA^®^, silicone injection
scomu2	*Stenella coeruleoalba* M., male newborn	Head coronal section
scomu3	*Stenella coeruleoalba* M., male juvenile	Head sagittal section
scomu4	*Stenella coeruleoalba* M., Male juvenile	Endoscopy
scomu5	*Stenella coeruleoalba* M., female adult	Head dissection, vascular latex injection
scomu6	*Stenella coeruleoalba* M., female adult	Head sagittal section, histological analysis
scomu7	*Stenella coeruleoalba* M., female adult	Head dissection
scomu8	*Stenella coeruleoalba* M., female adult	CT, AMIRA^®^, silicone injection
ecal1	*Equus caballus* L., male, fetus	Head dissection
ecal2	*Equus caballus* L., adult	Head dissection
ecal3	*Equus caballus* L., adult	Head section
ecal4	*Equus caballus* L., adult	Head section

DDE: *Delphinus delphis* from Galicia, Spain; SCOP: *Stenella coeruleoalba* from Galicia, Spain; SCOCE: *Stenella coeruleoalba* from Ceuta, Spain; SCOMU: *Stenella coeruleoalba* from Murcia, Spain; MRI: Magnetic resonance imaging; CT: Computed Tomography, CEMMA: Coordinator Center for the study of the marine mammals, Galicia; CECAM: Center for the study and conservation of marine animals, Ceuta; CRFS: Wildlife rehabilitation Center, Murcia; ECAL: *Equus caballus* from Alicante, Spain; AMIRA^®^ for FEI systems is a software platform for 3D and 4D data visualization, processing and analysis.

## Data Availability

Not applicable.

## Supplementary Materials

The following are available online at https://www.mdpi.com/2076-2615/11/2/441/s1, Table S1: Other parameters observed in this study, Table S2: MRI parameters used in this study.
