# Supplementary material for: Comparative Anatomy of the Nasal Cavity in the Common Dolphin Delphinus delphis L., Striped Dolphin Stenella coeruleoalba M. and Pilot Whale Globicephala melas T.: A Developmental Study
Source: Animals (Basel). 2021 Feb 8;11(2):441. doi: 10.3390/ani11020441 (PMC7915504; doi:10.3390/ani11020441)
Supplement: Supplementary file 1 [file animals-11-00441-s001.zip › Table S1.docx]

**Table S1.** Other parameters observed in this study.

| **Stranding Reference** | **Length, Weight, Estimated Gestation Time and Stranding Year [29, 39-40]** | **Preservation Techniques** |
| --- | --- | --- |
| DDE1,  CEMMA | 11.8 cm, 15.4 gr, 1,5 months approx., 2012. | Fixation: formaldehyde 10% and refrigeration |
| DDE2  CEMMA | 27.5 cm, 212.8 gr, 3,5 months approx., 2012. | Fixation: formaldehyde 10% and refrigeration |
| DDE3  CEMMA | 31 cm, 340.4 gr, 4 months aprox., 2014. | Fixation: formaldehyde 10% and refrigeration |
| SCOP1  CEMMA | 32.5 cm, 372 gr, 4.5 months approx., 2004. | Fixation: formaldehyde 10% and refrigeration |
| GMA1  CECAM | 40 cm, 628.3 gr, 5 months approx., 2013. | Fixation: formaldehyde 10% and refrigeration |
| DDE4  CEMMA | 41.5 cm, 842.3 gr, 5.5 months approx., 2014. | Fixation: formaldehyde 10% and refrigeration |
| DDE5  CEMMA | 41.7 cm, 655.3 gr, 5.5 months approx., 2009. | Fixation: formaldehyde 10% and refrigeration |
| DDE6  CEMMA | 44 cm, 864.5 gr, 5.8 months approx., 2008. | Fixation: formaldehyde 10% and refrigeration |
| DDE7  CEMMA | 44 cm, 1061.4 gr, 6 months approx., 2009. | Fixation: formaldehyde 10% and refrigeration |
| DDE8  CEMMA | 47.5 cm bis, 948.9 gr, 6 months approx., 2014. | Fixation: formaldehyde 10% and refrigeration |
| DDE9  CEMMA | 50.5 cm, 1037.4 gr, 7 months approx., 2012. | Fixation: formaldehyde 10% and refrigeration |
| DDE10  CEMMA | 50.5 cm, 1231.1 gr, 7.5 months approx., 2014. | Fixation: formaldehyde 10% and refrigeration |
| DDE11  CEMMA | 56 cm, 1470 gr, 8 months approx., 2009. | Fixation: formaldehyde 10% and refrigeration |
| DDE12  CEMMA | 58 cm, 1483.9 gr, 8,5months approx., 2008. | Fixation: formaldehyde 10% and refrigeration |
| DDE13  CEMMA | 65 cm, 3090 gr, 9 Months approx., 2004. | Embalming: formaldehyde, glycerine, isopropyl alcohol, phenol and refrigeration |
| DDE14  CEMMA | 75 cm, 3110 gr, 10 months, 2014. | Embalming: formaldehyde, glycerine, isopropyl alcohol, phenol and refrigeration |
| SCOCE1  CECAM | 85 cm, 9200 gr, 2014. | Fixation: formaldehyde 10% |
| SCOMU1  CRFS | 96 cm, 10.840 gr, 2017. | Fixation: formaldehyde 10% and freezing - 20ºC |
| SCOMU2  CFRS | 102 cm, 14.000 gr, 2012. | Fixation: formaldehyde 10% and refrigeration |
| SCOMU3  CFRS | 123 cm, 23.650 gr., 2019. | Fixation: formaldehyde 10% and refrigeration |
| SCOMU4  CFRS | 176 cm, 54,900 gr, 2019. | Freezing - 20ºC |
| SCOMU5  CRFS | 191 cm, 57,850 gr., 2017. | Embalming: formaldehyde, glycerine, isopropyl alcohol, phenol and refrigeration |
| SCOMU6  CFRS | 201 cm, 83.850 gr, 2018. | Embalming: formaldehyde, glycerine, isopropyl alcohol, phenol and refrigeration |
| SCOMU7  CFRS | 186 cm, 84.000 gr, 2017 | Refrigeration |
| SCOMU8  CRFS | 206 cm, 77.000 gr, 2018 | Fixation: formaldehyde 10% and refrigeration |
| ECAL1 | 28 cm, 354 gr, - | Fixation: formaldehyde 10% |
| ECAL2 | - | Embalming: formaldehyde, glycerine, isopropyl alcohol, phenol and refrigeration |
| ECAL3 | - | Embalming: formaldehyde, glycerine, isopropyl alcohol, phenol and refrigeration |
| ECAL3 | - | Embalming: formaldehyde, glycerine, isopropyl alcohol, phenol and refrigeration |
|  |  |  |

*DDE: Delphinus delphis* from Galicia, Spain*; SCOP*: *Stenella coeruleoalba* from Galicia, Spain; *SCOCE*: *Stenella coeruleoalba* from Ceuta, Spain; *SCOMU*: *Stenella coeruleoalba* from Murcia, Spain; MRI: Magnetic resonance imaging; CT: Computed Tomography, *CEMMA*: Coordinator Center for the study of the marine mammals, Galicia; *CECAM*: Center for the study and conservation of marine animals, Ceuta; *CRFS*: Wildlife rehabilitation Center, Murcia; ECAL: *Equus caballus* from Alicante, Spain.
