# Supplementary material for: Comparative Anatomy of the Nasal Cavity in the Common Dolphin Delphinus delphis L., Striped Dolphin Stenella coeruleoalba M. and Pilot Whale Globicephala melas T.: A Developmental Study
Source: Animals (Basel). 2021 Feb 8;11(2):441. doi: 10.3390/ani11020441 (PMC7915504; doi:10.3390/ani11020441)
Supplement: Supplementary file 1 [file animals-11-00441-s001.zip › Table S2.docx]

**Table S2.** MRI parameters used in this study.

| **Study code** | **Weighted** | **Pulse sequence** | **Dimensional plane** | **Acquisition** | **TE** | **TR** | **TI** | **NEX** | **Slice thickness** | **Interslice gap** | **Field of view** | **Matrix dimensions** |
| --- | --- | --- | --- | --- | --- | --- | --- | --- | --- | --- | --- | --- |
| dde1 | T1 | se | sagittal | 2D | 16 | 260 | 0 | 3 | 1 | 1.2 | 100 | 0\320\224\0 |
| dde1 | T1 | se | coronal | 2D | 13 | 400 | 0 | 3 | 1 | 1.1 | 100 | 0\256\192\0 |
| dde1 | T2 | frfse | coronal | 2D | 114 | 2160 | 0 | 4 | 1 | 1.2 | 100 | 0\320\224\0 |
| dde2 | T2 | frfse | sagittal | 2D | 104 | 2000 | 0 | 3 | 3 | 3.3 | 100 | 0\320\224\0 |
| dde3 | T1 | se | sagittal | 2D | 14 | 360 | 0 | 3 | 3 | 3.3 | 100 | 0\320\224\0 |
| dde3 | T2 | frfse | sagittal | 2D | 104 | 2000 | 0 | 3 | 3 | 3.3 | 100 | 0\320\224\0 |
| dde3 | T1 | se | coronal | 2D | 10 | 420 | 0 | 3 | 3 | 3.3 | 100 | 0\320\224\0 |
| dde3 | T2 | frfse | coronal | 2D | 104 | 2500 | 0 | 3 | 3 | 3.3 | 100 | 0\320\224\0 |
| scop1 | T1 | se | sagittal | 2D | 14 | 500 | 0 | 0.5 | 3 | 3.3 | 100 | 0\256\224\0 |
| scop1 | T2 | frfse | sagittal | 2D | 112 | 3460 | 0 | 2 | 3 | 3.3 | 100 | 256\0\0\192 |
| scop1 | T1 | se | coronal | 2D | 14 | 220 | 0 | 0.5 | 3 | 3.3 | 100 | 0\256\192\0 |
| scop1 | T2 | frfse | coronal | 2D | 86 | 2140 | 0 | 2 | 3 | 3.1 | 100 | 0\320\224\0 |
| gma1 | T1 | se | sagittal | 2D | 14 | 400 | 0 | 3 | 3 | 3.3 | 100 | 0\320\224\0 |
| gma1 | T2 | frfse | sagittal | 2D | 104 | 2000 | 0 | 2 | 3 | 3.3 | 100 | 0\320\224\0 |
| gma1 | T1 | se | coronal | 2D | 10 | 540 | 0 | 3 | 3 | 3.3 | 100 | 0\320\224\0 |
| gma1 | T2 | frfse | coronal | 2D | 104 | 3240 | 0 | 3 | 3 | 3.3 | 100 | 0\320\224\0 |
| dde5 | T2 | frfse | sagittal | 2D | 104 | 2000 | 0 | 3 | 3 | 3.3 | 100 | 0\320\224\0 |
| dde7 | T1 | se | sagittal | 2D | 14 | 220 | 0 | 3 | 4 | 4.1 | 75 | 0\320\224\0 |
| dde7 | T2 | frfse | sagittal | 2D | 111 | 2240 | 0 | 2 | 4 | 4.1 | 100 | 384\0\0\224 |
| dde7 | T1 | se | coronal | 2D | 12 | 460 | 0 | 2 | 3.5 | 3.8 | 75 | 0\256\256\0 |
| dde7 | T2 | frfse | coronal | 2D | 12 | 460 | 0 | 2 | 3.5 | 3.8 | 75 | 0\256\256\0 |
| dde9 | T1 | se | sagittal | 2D | 14 | 300 | 0 | 3 | 4 | 4.1 | 75 | 0\320\224\0 |
| dde9 | T2 | frfse | sagittal | 2D | 109 | 2700 | 0 | 2 | 4 | 4.1 | 100 | 384\0\0\224 |
| dde9 | T1 | se | coronal | 2D | 14 | 300 | 0 | 1 | 3.5 | 3.8 | 75 | 0\320\256\0 |
| dde9 | T2 | frfse | coronal | 2D | 12 | 460 | 0 | 2 | 3.5 | 3.8 | 75 | 0\256\256\0 |
| dde10 | T1 | se | sagittal | 2D | 14 | 300 | 0 | 3 | 3 | 3.3 | 100 | 0\320\224\0 |
| dde10 | T2 | frfse | sagittal | 2D | 104 | 3000 | 0 | 3 | 3 | 3.3 | 100 | 0\320\224\0 |
| dde10 | T1 | se | coronal | 2D | 10 | 300 | 0 | 3 | 3 | 3.3 | 100 | 0\320\224\0 |
| dde10 | T2 | frfse | coronal | 2D | 104 | 1760 | 0 | 3 | 3 | 3.3 | 100 | 0\320\224\0 |
| dde11 | T1 | frfse | sagittal | 2D | 58 | 2.1 | 0 | 1 | 5 | 10 | 100 | 0\256\128\0 |
| dde11 | T2 | Frfse | sagittal | 2D | 104 | 2000 | 0 | 3 | 3 | 3.3 | 100 | 0\320\224\0 |
| dde11 | T1 | se | coronal | 2D | 10 | 300 | 0 | 2 | 3 | 3.3 | 100 | 0\320\224\0 |
| dde11 | T2 | frfse | coronal | 2D | 104 | 3500 | 0 | 3 | 3 | 3.3 | 100 | 0\320\224\0 |
| dde12 | T1 | se | sagittal | 2D | 14 | 300 | 0 | 3 | 4 | 4.1 | 75 | 0\320\224\0 |
| dde12 | T2 | frfse | sagittal | 2D | 109 | 2920 | 0 | 2 | 4 | 4.1 | 100 | 384\0\0\224 |
| dde12 | T1 | se | coronal | 2D | 12 | 300 | 0 | 1 | 3.5 | 3.8 | 75 | 0\256\192\0 |
| dde12 | T2 | frfse | coronal | 2D | 86 | 2700 | 0 | 1 | 3.5 | 3.8 | 75 | 0\320\224\0 |
| dde13 | T1 | se | sagittal | 2D | 14 | 300 | 0 | 3 | 4 | 4.1 | 75 | 0\320\224\0 |
| dde13 | T2 | frfse | sagittal | 2D | 105 | 3820 | 0 | 2 | 4 | 4.1 | 100 | 384\0\0\224 |
| dde13 | T1 | se | coronal | 2D | 14 | 460 | 0 | 2 | 3.5 | 3.8 | 75 | 0\320\256\0 |
| dde13 | T2 | frfse | coronal | 2D | 94 | 4040 | 0 | 2 | 3.5 | 3.8 | 75 | 0\320\224\0 |
| dde14 | T1 | se | sagittal | 2D | 14 | 300 | 0 | 3 | 4 | 4.1 | 75 | 0\320\224\0 |
| dde14 | T2 | frfse | sagittal | 2D | 107 | 3500 | 0 | 2 | 4 | 4.1 | 100 | 384\0\0\224 |
| dde14 | T1 | se | coronal | 2D | 14 | 240 | 0 | 2 | 3.5 | 3.8 | 75 | 0\320\256\0 |
| dde14 | T2 | frfse | coronal | 2D | 92 | 3200 | 0 | 2 | 3.5 | 3.8 | 75 | 0\320\224\0 |

*se:* Spin echo sequence; *FrFse:* Fast Recovery Fast Spin Echo sequence.
